# Variation in body size and sexual size dimorphism in the most widely ranging lizard: testing the effects of reproductive mode and climate

**DOI:** 10.1002/ece3.6077

**Published:** 2020-05-06

**Authors:** Evgeny S. Roitberg, Valentina F. Orlova, Nina A. Bulakhova, Valentina N. Kuranova, Galina V. Eplanova, Oleksandr I. Zinenko, Oscar Arribas, Lukáš Kratochvíl, Katarina Ljubisavljević, Vladimir P. Starikov, Henk Strijbosch, Sylvia Hofmann, Olga A. Leontyeva, Wolfgang Böhme

**Affiliations:** ^1^ Zoological Research Museum Alexander Koenig Bonn Germany; ^2^ Zoological Research Museum Moscow M.V. Lomonosov State University Moscow Russia; ^3^ Institute of Biological Problems of the North Magadan Russia; ^4^ Research Institute of Biology and Biophysics Tomsk State University Tomsk Russia; ^5^ Department of Vertebrate Zoology and Ecology Tomsk State University Tomsk Russia; ^6^ Institute of Ecology of the Volga River Basin Togliatti Russia; ^7^ Museum of Nature V. N. Karazin Kharkiv National University Kharkiv Ukraine; ^8^ Avda. Francisco Cambó 23, 08003 Barcelona Spain; ^9^ Department of Ecology Charles University Prague Czech Republic; ^10^ Institute for Biological Research University of Belgrade Belgrade Serbia; ^11^ Department of Biology and Biotechnology Surgut State University Surgut Russia; ^12^ RAVON Radboud University BK Nijmegen The Netherlands; ^13^ Helmholtz‐Centre for Environmental Research – UfZ Leipzig Germany; ^14^ Department of Biogeography Moscow M. V. Lomonosov State University Moscow Russia

**Keywords:** Bergmann's rule, ecogeographic body size clines, life‐history, lizards, Rensch's rule, *Zootoca vivipara*

## Abstract

Reproductive mode, ancestry, and climate are hypothesized to determine body size variation in reptiles but their effects have rarely been estimated simultaneously, especially at the intraspecific level. The common lizard (*Zootoca vivipara*) occupies almost the entire Northern Eurasia and includes viviparous and oviparous lineages, thus representing an excellent model for such studies. Using body length data for >10,000 individuals from 72 geographically distinct populations over the species' range, we analyzed how sex‐specific adult body size and sexual size dimorphism (SSD) is associated with reproductive mode, lineage identity, and several climatic variables. Variation in male size was low and poorly explained by our predictors. In contrast, female size and SSD varied considerably, demonstrating significant effects of reproductive mode and particularly seasonality. Populations of the western oviparous lineage (northern Spain, south‐western France) exhibited a smaller female size and less female‐biased SSD than those of the western viviparous (France to Eastern Europe) and the eastern viviparous (Eastern Europe to Far East) lineages; this pattern persisted even after controlling for climatic effects. The phenotypic response to seasonality was complex: across the lineages, as well as within the eastern viviparous lineage, female size and SSD increase with increasing seasonality, whereas the western viviparous lineage followed the opposing trends. Altogether, viviparous populations seem to follow a saw‐tooth geographic cline, which might reflect the nonmonotonic relationship of body size at maturity in females with the length of activity season. This relationship is predicted to arise in perennial ectotherms as a response to environmental constraints caused by seasonality of growth and reproduction. The SSD allometry followed the converse of Rensch's rule, a rare pattern for amniotes. Our results provide the first evidence of opposing body size*—*climate relationships in intraspecific units.

## Graphical abstract

We analyzed the association of adult body size and sexual size dimorphism (SSD) with reproductive mode (viviparity vs. oviparity), lineage identity, and climate in the common lizard (*Zootoca vivipara*), the species occupying almost entire Northern Eurasia. We revealed a moderate effect of reproductive mode (larger female size and higher SSD in viviparous vs. oviparous populations) and a strong but complex effect of seasonality: female size and SSD decrease with increasing seasonality in the Western viviparous lineage distributed from France to Eastern Europe, while the Eastern viviparous lineage ranging from Eastern Europe to the Far East showed the opposite trends. Our results provide the first evidence of opposing body size—climate relationships in clearly intraspecific units.

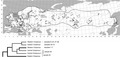

## 1 INTRODUCTION

The patterns and causes of geographic variation in body size are fundamental themes in studies on life‐history evolution (Angilletta, Niewiarowski, Dunham, Leaché, & Porter, 2004; Arendt & Fairbairn, 2012; Roff, 2002). Their importance has further increased in connection with the ongoing climate change, as trends in space may be highly relevant for predictions of changes over time (Gardner, Peters, Kearney, Joseph, & Heinsohn, 2011; Millien et al., 2006; Teplitsky & Millien, 2014). Yet, despite the growing body of publications, the diversity of ecogeographic body size clines remains not fully understood, particularly in ectotherms (see Angilletta, Niewiarowski, et al., 2004; Blanckenhorn & Demont, 2004; Hjernquist et al., 2012; Rypel, 2014; Sears & Angilletta, 2004 for important advances).

Latitudinal and altitudinal clines in body size are the most widely observed ecogeographic patterns (e.g., Ashton & Feldman, 2003; Blanckenhorn & Demont, 2004). Temperature is often assumed to be the principal determinant of ecogeographic body size clines, because temperature covaries consistently with latitude and altitude, and it strongly affects vital processes in the organisms (Angilletta, 2009). Yet, a number of recent studies have found that water availability (precipitation, humidity), and particularly seasonality (within‐year variation in temperature or precipitation), often explain a higher proportion of body size variation than does mean temperature (e.g., Ashton, 2001; Çağlar, Karacaoğlu, Kuyucu, & Sağlam, 2014; Stillwell, Morse, & Fox, 2007). For each of these factors, multiple mechanistic hypotheses have been proposed (see below). However, rigorous testing of such hypotheses is often impeded by collinearity between climatic variables (Millien et al., 2006) which is particularly common within limited geographic areas. Specifically, colder environments are often associated with a greater seasonality (Aragón & Fitze, 2014; Chown & Klok, 2003; Körner, 2000). Furthermore, the pattern of the relationship between a phenotypic trait and a climatic covariate can be nonmonotonic, such as inverted U clines (Hjernquist et al., 2012) or saw‐tooth patterns (Masaki, 1967; Mousseau, 1997). Yet, such more complex patterns are unlikely to be revealed within limited spatial and environmental ranges (Ashton & Feldman, 2003; Gaston, Chown, & Evans, 2008).

Wide‐ranging species present promising models for studying phenotype*—*climate relationships, because the variation of target traits can be documented for numerous geographically distinct populations exhibiting a wide range of climates and diverse combinations of putative predictors (Roitberg et al., 2013, 2015; *cf*. Meiri, Yom‐Tov, & Geffen, 2007; Jetz, Ashton, & La Sorte, 2009). The problem is that wide‐ranging species often consist of several phylogeographic lineages. Pooling samples from different lineages may lead to a spurious trait‐climate correlation (Romano & Ficetola, 2010) or obscure true relationships (Ashton, 2001). Therefore, even though body size is both phenotypically plastic and evolutionary malleable (Falconer, 1989; Green & Middleton, 2013; Jetz et al., 2009; Millien et al., 2006), the effects of current environment should be examined jointly with those of ancestry (Ashton, 2004; Diniz‐Filho, 2008; Gaston et al., 2008). Yet, comprehensive range‐wide studies of this kind have rarely been conducted on widespread species, even for fundamentally important traits such as body size (Angilletta, Niewiarowski, et al., 2004; Horváthová et al., 2013; Roitberg et al., 2013). Furthermore, although studies of intraspecific body size variation usually consider both sexes, they seldom explore how body size differences between males and females (i.e., sexual size dimorphism, SSD) vary along geographic or climatic gradients (Laiolo, Illera, & Obeso, 2013; Litzgus & Smith, 2010; Roitberg, 2007; Roitberg et al., 2015; Stillwell et al., 2007). The latter aspect is important because due to sexual differences in reproductive and ecological roles, some factors can affect one sex more than the other. As a result, geographic patterns in body size may differ markedly between males and females (Herczeg, Gonda, & Merilä, 2010; Pearson, Shine, & Williams, 2002; Roitberg et al., 2015; Saino & De Bernardi, 1994; Thorpe & Baez, 1987).

**Figure 1:**
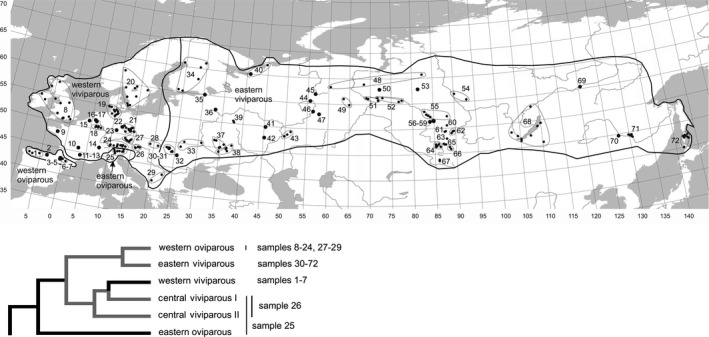
Geographic ranges of different clades of *Zootoca vivipara*, their phylogenetic relationships (after Surget‐Groba et al., 2006), and our study sites. Details for study samples (1–72) are given in Appendix A7 (Table A1). Two relic lineages (central viviparous I and II), sporadically distributed in the south of Central Europe, are not shown. Note that the distribution border of the eastern oviparous clade (*Z. v. carniolica*) is actually jagged so that samples 25 and 26 may represent mixtures of different clades (W. Mayer, unpublished data; see also Cornetti et al., 2015; Lindtke et al., 2010) and are excluded from our analyses

The European common lizard (*Zootoca vivipara*), one of the most widely distributed terrestrial reptile in the world, is an excellent model for such studies. It occupies almost the entire Northern Eurasia and includes several viviparous and oviparous lineages (clades), three of them inhabiting wide ranges of climates (Figure 1). For this species, there is range‐wide phylogeographic analysis (Surget‐Groba et al., 2006) and extensive data on body size and other life‐history traits for multiple populations (e.g., Bauwens & Verheyen, 1987; Heulin, 1985; Pilorge, 1987). Furthermore, *Z. vivipara* has become a model species for observational (Chamaillé‐Jammes, Massot, Aragón, & Clobert, 2006; Le Galliard, Marquis, & Massot, 2010; Rutschmann et al., 2016) and experimental (Bestion, Teyssier, Richard, Clobert, & Cote, 2015) studies on how life‐history phenotype may respond to ongoing climate warming. Roitberg et al. (2012, 2013) and Horváthová et al. (2013) studied geographic variation of several life‐history traits in *Z. vivipara*. However, these studies considered only female size, and they covered the large eastern part of the species range poorly.

The aim of our study was to compile a comprehensive set of body size data for *Z. vivipara* across Eurasia and estimate the effects of reproductive mode and lineage identity, and the effects of climate on adult body size and sexual size dimorphism in this species. Our specific hypotheses and their predictions are summarized in Table 1 and presented in detail below. Other things being equal, we consider explanations based on plasticity as more parsimonious than hypotheses implying genetic adaptation (Chown & Klok, 2003; Madsen & Shine, 1993; Roitberg et al., 2013).

**Table 1:** Summary of specific hypotheses and their predictions tested in our study

Factor	Proxy	Phenotypic response	Suggested mechanism
Thermal regime during activity season	Mean summer temperature (T2)	Body size increases with T2 (**Prediction 1**)	Heat acquisition hypothesis^a^
		Body size decreases with T2 (**Prediction 2**)	(1) Heat conservation hypothesis^a^ (2) Temperature‐size rule^b^
Hydric regime during activity season	Summer precipitation (P2)	Body size decreases with P2 (**Prediction 3**)	(1) Dehydration resistance hypothesis^a^ (2) Immediate negative effect of rainfall on lizard activity, food intake, and hence on body growth^b^
		Body size increases with P2 (**Prediction 4**)	Delayed positive effect of rainfall on habitat quality, including food availability^b^
Length of activity season	Seasonality (here, Mean winter temperature, T1)	Body size increases with T1 (*converse pseudo‐Bergmann's cline*, **Prediction 5**)	Adolph and Porter (1993) “null model” (age at maturity is constant)^b^
		Body size decreases with T1 (*pseudo‐Bergmann's cline*, **Prediction 6**)	(1) Adolph and Porter (1996) “main model” (modal age at maturity shifts abruptly as season length reaches a threshold)^b^ (2) Starvation resistance hypothesis^a^
Sex‐specific effects of cold or seasonal climate	T2 or T1	Larger female size and converse Rensch's allometry of SSD (**Prediction 7a**)^c^	Cold or seasonal climates reduce reproduction frequency, selecting for larger female size^a^
		Smaller male size and standard Rensch's allometry of SSD (**Prediction 7b**)^a^, ^d^	Cold or seasonal climates exert energetic constraints on growth and aggressive behavior, thus selecting for smaller male size^a^
Sex‐specific effects of reproductive mode	Oviparous versus viviparous clades	Female size and SSD larger in viviparous forms (**Prediction 8**)^a^, ^e^	Viviparity is associated with: (1) lower reproduction frequency; (2) higher gestation costs; (3) stronger maternal body‐volume constraints on reproductive output

Note

See text for details and references.

Abbreviation: SSD, sexual size dimorphism.

a Hypotheses based on genetic adaptation.

b Hypotheses based on plasticity.

c Adolph & Porter's “main model” actually predicts a marked decrease of body size with T1 around the threshold values resulting in a *saw‐tooth cline* whose overall linear trend is decreasing body size with T1.

d Female size varies more than male size among populations.

e Male size varies more than female size among populations.

### 1.1 Hypotheses related to temperature

While larger size reduces the surface‐to‐volume ratio, thus better conserving the heat (Bergmann, 1847), smaller size allows getting external heat rapidly (Ashton & Feldman, 2003 and references therein). The latter consideration, which we term *heat acquisition hypothesis*, is widely accepted for terrestrial ectotherms (Ashton & Feldman, 2003; Oufiero, Gartner, Adolph, & Garland, 2011; Pianka & Vitt, 2003; Pincheira‐Donoso, Hodgson, & Tregenza, 2008). It predicts a converse Bergmann cline, that is, a positive correlation between body size and mean ambient temperature (**Prediction 1**). Bergmann's (1847) *heat conservation hypothesis*, predicting a negative correlation between body size and mean ambient temperature (a “standard” Bergmann cline; **Prediction 2**), is often considered poorly relevant to ectotherms (Cushman, Lawton, & Manly, 1993; Pincheira‐Donoso et al., 2008). Both thermoregulation‐related hypotheses are clearly selectionistic, that is, imply genetic adaptation (e.g., Adams & Church, 2011; Litzgus, DuRant, & Mousseau, 2004).

An alternative but not mutually exclusive hypothesis for Bergmann's clines is the *temperature‐size rule*, a predominant pattern of developmental plasticity in ectotherms. This rule postulates that individuals growing at lower temperature mature later but at a larger size than conspecifics growing at higher temperature (Angilletta, 2009; Atkinson, 1994).

### 1.2 Hypotheses related to water availability

Considering that large individuals have a reduced surface‐to‐volume ratio and overall higher absolute water content compared to small individuals, an adaptive *dehydration resistance hypothesis* predicts a negative correlation of body size with precipitation or humidity (**Prediction 3**) (see Stillwell et al., 2007 for references). Summer precipitation may also directly affect growth and body size in ectotherms; specifically in lizards and insects, there can be immediate, negative effects on insolation and consequently on animal's activity, food intake and thus body growth and positive, delayed effects on habitat productivity, which enhance foraging opportunity of lizards in later times (reviewed by Çağlar et al., 2014; Le Galliard et al., 2010). The negative effects correspond to our Prediction 3, while the positive ones to **Prediction 4**.

### 1.3 Hypotheses related to seasonality

Among multiple hypotheses relating body size variation to the length of annual activity (or inactivity) the models of Adolph and Porter (1993, 1996) seem particularly relevant for our study since they were developed specifically for lizards as perennial terrestrial ectotherms with advanced behavioral thermoregulation. The basic point of their reasoning is that annual growth increment in such organisms is mainly determined by the *length of activity season* rather than *environmental temperature*. Their "null physiological model", that is, the basic model assuming the absence of other factors, predicts smaller body size in more seasonal climates (**Prediction 5**; Adolph & Porter, 1993) which reduce energy acquisition opportunities. This pattern can be termed “converse *pseudo‐*Bergmann's cline” to distinguish from the *true* Bergmann's and *true* converse Bergmann's clines which relate to environmental temperature.

The “main” model by Adolph and Porter (1996) additionally considers a discontinuous variation in the age at maturity (the age at the first reproduction), which is inherent to perennial ectotherms in seasonal climates. It predicts that this variation may reverse the body size cline expected by the null model. As the length of activity season decreases below some threshold, the modal group of subadult individuals cannot attain an appropriate body size within the reproductive season of the same year of life as their conspecifics in an environment allowing longer activity season. Under such constraints, the subadults invest available energy into further growth and start reproduction in the following season. This *disproportional* prolongation of juvenile growth may overcompensate the shortening of the annual activity period and enhance the typical size at maturity, at least within some range of climates. The predicted pattern is increasing adult body size in more seasonal climates (“*pseudo‐*Bergmann's” cline; **Prediction 6**). The main model predicts a *saw‐tooth cline* (Adolph & Porter, 1996: Figure 4) whose overall linear trend is likely a pseudo‐Bergmann cline as well. As reproduction is expected to more strongly inhibit body growth in females than in males, earlier maturation might be responsible for smaller female relative to male size in warmer or less seasonal climates (see Roitberg & Smirina, 2006 for indirect evidence in another lacertid lizard, *Lacerta agilis*). Thus, specifically the effect predicted by the main model may be female‐biased. Note that the underlying mechanism of Adolph & Porter's models is a direct response to environmental constraints which is not necessarily accompanied by genetic divergence.

Prediction 6 is also made by the adaptive *fasting endurance*, or *starvation resistance* hypothesis (e.g., Aragón & Fitze, 2014; Ficetola et al., 2010). Its version that applies to temperate zone reptiles explains pseudo*‐*Bergmann clines via ability of larger‐sized animals to acquire and carry larger fat reserves relative to metabolic needs than smaller‐sized animals, this advantage being more important in climates with longer winters (Ashton, 2001; Litzgus et al., 2004).

### 1.4 Hypotheses related to sex‐specific selection or plasticity

Two distinct hypotheses related to sex‐specific selection predict more female‐biased SSD in colder and more seasonal climates. The extended *fecundity‐advantage hypothesis* (reviewed by Cox, Skelly, & John‐Alder, 2003; see also Angilletta, Steury, & Sears, 2004; Litzgus & Smith, 2010; Roitberg et al., 2015) suggests that reduced reproduction frequency should select for higher fecundity and thus for larger females (**Prediction 7a**).

The *small male advantage hypothesis* (reviewed by Blanckenhorn, 2000, 2005; Zamudio, 1998; Cox et al., 2003) argues that at low population densities the importance of male–male agonistic interactions, which select for larger body size, should decrease, while the disadvantage of lower mobility (which is often associated with large size) should increase. This hypothesis can be extended for a wider range of conditions. For instance in ectotherms, cold or highly seasonal climates, which reduce energy acquisition opportunities (Congdon, 1989), should also select against energetically costly aggressive behavior (and decrease the benefits of large male size) while increasing the small size‐associated advantage of lower resource demands. Thus, we predict smaller male size in cold or highly seasonal climates (**Prediction 7b**).

Three related hypotheses predict larger female size and stronger SSD in viviparous versus oviparous forms (**Prediction 8**). First, like colder environments, viviparity should reduce reproduction frequency, thus selecting for larger females (the extended *fecundity‐advantage hypothesis*, reviewed by Cox et al., 2003). Second, viviparity may favor larger females via strengthening the maternal body‐volume constraints on reproductive output (reviewed by Roitberg et al., 2013). Third, viviparity enhances costs of pregnancy involving not only physical burden but also metabolic costs (Bleu, Massot, Haussy, & Meylan, 2012; Foucart, Lourdais, DeNardo, & Heulin, 2014; Guillette, 1982). These costs include a marked fecundity‐independent component (Foucart et al., 2014) which may confer additional, *survival* advantage to larger females (*cf*. Madsen & Shine, 1994).

Besides sex‐specific selection, ecogeographic clines in SSD may reflect *sex‐differential plasticity* (Cox & Calsbeek, 2010; Fairbairn, 2005; Hu, Xie, Zhu, Wang, & Lei, 2010; Madsen & Shine, 1993). Albeit the latter hypothesis predicts no particular pattern of body size*—*climate relationship, it may contribute to explaining an SSD cline when proximate mechanisms of SSD are known or inferable (see Section 4). An important descriptive aspect of body size variation is SSD allometry. This allometry either follows Rensch's rule (male size varies more than female size among populations, resulting in slopes greater than one when log[male size] is regressed on log[female size]) or the opposite, converse Rensch's, pattern (Blanckenhorn, Stillwell, Young, Fox, & Ashton, 2006; Fairbairn, 1997). Specifically, Prediction 7b implies a Rensch's rule, while Predictions 7a and 8 imply its converse.

## 2 MATERIAL AND METHODS

### 2.1 Study species

*Zootoca vivipara* is a small (adult snout‐vent length 40–80 mm), ground‐dwelling, insectivorous, heliothermic lizard. Compared to most other lizards *Z. vivipara* shows a high resistance to low temperatures and a low resistance to desiccation (Reichling, 1957). It prefers humid habitats, mostly in the forest vegetation zone (see Figure 1 and Roitberg et al., 2013 for further details and references).

### 2.2 Body size data

We used the snout‐vent length (SVL), the primary measure of body size in lizards and snakes (Roitberg et al., 2011 and references therein), as a proxy for overall structural size. We summarized original and published SVL data for 19,935 common lizards from 240 localities combined in 72 geographically distinct study samples (populations); these cover a major part of the species range (Figure 1; Appendix A7: Table A1). The original data come from museum samples or from previous studies mostly performed for parasitological monitoring (Kuranova et al., 2011). Additional data were extracted from published histograms (e.g., Pilorge, 1987); in few cases, we also considered summary statistics for sex‐specific adult SVL. When data for the same site were found in several studies, all unique samples for each sex were included to increase sample size. In total, 5,055 males and 6,474 females were considered as adults and constituted our study samples (see below).

### 2.3 Data analysis

Within localities, samples from different years were pooled to increase sample sizes and to apply a standard approach across all data. Whenever reasonable sample sizes were available we used strictly local samples, both for original and published data. When local sample sizes were too small, however, we pooled them into compound samples for larger geographic areas (Figure 1) and used in our analyses weighted means for the study traits and nonweighted means for climatic variables. See Roitberg et al. (2013) for our criteria for pooling samples.

To test robustness of our analyses of the variation in adult body size and SSD to potential confounding factors (see Section 2.4 below) all main analyses were performed for two sets of data. Data set 1 included the animals assigned to adults by the primary researcher (Appendix A7: Table A1, *A*). Data set 2 was based on single inclusion criteria across samples: body length equal to or exceeding 45 mm for males and 48 mm for females (Table A1, *B*). These thresholds are close to typical minimum SVL of mature common lizards reported for most viviparous (Avery, 1975; Cavin, 1993; Orlova, 1975; Pilorge & Xavier, 1981) and some oviparous (Sinervo et al., 2007) populations studied. Furthermore, each main analysis was run for mean values and additionally for the 80th percentiles of the size distributions (see Roitberg, 2007 for details and references on higher percentiles as useful estimators of population's typical adult body size in indeterminate growers; see also Case, 1976). Thus, we used four metrics for each of our target traits, that is, male size, female size, and SSD. Means and percentiles of sex‐specific SVL were ln‐transformed for all analyses except SSD.

Sexual size dimorphism was quantified with the index: *SDI* = (*size of larger sex*/*size of smaller sex*)* − 1*, conventionally expressed as positive if females are larger and negative if males are larger (Lovich & Gibbons, 1992). This index shows several favorable properties (Lovich & Gibbons, 1992; Smith, 1999) and has become a standard metric for studies on sexual dimorphism (Fairbairn, Blanckenhorn, & Szekely, 2007). SSD allometry was quantified with the slope of major axis regression (model II) of log(male SVL) on log(female SVL) (Fairbairn, 1997). The slopes (*β*) and their 95% confidence intervals were computed with the *lmodel2* package (Legendre, 2013) in R (R Core Team, 2018). They were tested against the null hypothesis of *β* = 1 (isometry). The pattern with *β* > 1 is referred to as Rensch's rule, and that with *β* < 1 as converse Rensch's allometry (Table 1).

The following bioclimatic indices were used as explanatory variables: mean temperature of coldest quarter (hereafter T1, winter temperature; Worldclim code BIO11), mean temperature of warmest quarter (T2, summer temperature; BIO10), and precipitation of warmest quarter (P2, summer precipitation; BIO18). T1 is a strong correlate of seasonality (Appendix A3), thus being a reasonable proxy for the *length* of activity season (Angilletta, Niewiarowski, et al., 2004); variation in T1 reflects the principal direction of climatic variation across temperate Eurasia (Appendix A2). T2 and P2 were our proxies respectively for *thermal conditions* and *water availability* during activity season, since the summer months fall into activity periods in virtually all populations. See Appendices A1, A2, A3 for further details on our climatic variables, their covariation patterns, as well as extraction of climate data.

The fourth explanatory variable was clade identity (western oviparous vs. western viviparous vs. eastern viviparous). See Appendix A4 for details and justification of a rough control for ancestry in this study. The effects of spatial autocorrelations and multicollinearity were considered as described in Appendices A5 and A6, respectively.

To simultaneously analyze categorical (clade identity, hereafter Clade) and continuous (climatic variables) effects, as well as their interactions, on the variation among population means (or percentiles) of a target trait, we used general linear models (GLMs). We determined the best combination of predictors of each target trait using an information‐theoretic approach (Burnham & Anderson, 2002) based on the Akaike's Information Criterion corrected for small sample sizes (AICc). Prior to model fitting, all continuous input variables were standardized to mean = 0 and *SD* = 1 to improve the interpretability of main effects in the presence of significant interactions (Schielzeth, 2010). We then fitted models encompassing all possible combinations of input variables and their first‐order interactions, including an intercept‐only model, calculating for each combination the AICc score. The interactions were included for explorative purposes. Models with ΔAICc ≤ 2 were considered candidate models and used for further analysis. As the Akaike's criterion may select overly complex models, we considered a complex model as a candidate model only if its AICc was lower than AICc of all simpler models nested in the complex model (Richards, Whittingham, & Stephens, 2011). Model selection was performed in R version 3.4.3 using the “MuMIn” package (Bartoń, 2017). Considering collinearity between Clade and T1 (Appendix A6) we refrained from model averaging, as recommended by Freckleton (2011). Instead, we summarized our candidate models verbally. We also performed some additional analyses exploring the effect of clade identity on "climate‐corrected" body size.

To evaluate the effect of reproductive mode the western oviparous clade (the only oviparous clade in our data set) was used as the reference level of the factor Clade. Furthermore, all GLM analyses were repeated for two viviparous clades only. Comparing the effects of Clade in the two data sets (three clades vs. two viviparous clades) provided additional evaluations of the effect of reproductive mode.

Values of all response and explanatory variables for 72 study samples are provided in Appendix A7 (Tables A1 and A2).

### 2.4 Methodological caveats

In species with continuing growth after maturity, numerous factors unrelated to geographic variation, such as local and temporal fluctuations in the abiotic (e.g., temperature and humidity) and/or biotic (e.g., food resources) environment, can affect patterns of growth, maturation, and survival of different cohorts and thus body size distribution in a particular study sample (Kratochvíl & Frynta, 2002; Roitberg, 2007; Shine, 1994, 2005; Stamps, 1993; Watkins, 1996). Further biases can come from compiling data of several independent researchers. They may differ in measuring routine, type of material (living vs. freshly euthanized vs. preserved specimens), and in their criteria of separating adults from immature animals, that is, inclusion criteria (these can be based e.g., on body size and color pattern vs. the state of gonads). The biases from the first two factors are expected to be within a few percents (Case, 1976; Roitberg et al., 2011; E. S. Roitberg, unpublished data; Vervust, Van Dongen, & Van Damme, 2009), and this is much lower than the observed variation within and among our study samples. Indeed, the factor Type of material was never significant when included in our best models as additional predictor. We also examined potential bias from temporal trends in adult body size (e.g., Chamaillé‐Jammes et al., 2006; Green & Middleton, 2013) by adding the factor Time (1950–1990 vs. 1991–2000 vs. 2001–2015); this addition did not improve our best models. The effects of inclusion criteria, and those of temporal variation in the proportion of newly matured animals (Watkins, 1996), were accounted for by using four different metrics for adult body size (see Section 2.3).

## 3 RESULTS

### 3.1 Candidate models for male size

Geographic variation in male SVL was weak (range of sample means 7 mm, min–max 48–55 mm; Appendix A7: Table A1) and poorly explained with our predictors. Even the AICc best‐fit models (hereafter top models) explain only 2%–6% of the total variance (Table 2), and in most analyses they do not perform considerably better than the intercept‐only model (ΔAICc ≤ 2). Only metric 4 explains 17% (Table 2, model 29) and only in one data set.

**Table 2:** AICc‐selected models (ΔAICc ≤ 2) for male size (SVL)

Metric	Model	*df*	AICc	ΔAICc	Weight	Formula	*R* ^2^	Adj *R^2^*	*p*
Three‐clade analyses									
M1	1	4	−270.34	0.00	0.167	T1 + 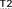	.079	.051	.067
M1	2	3	−270.31	0.03	0.164	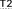	.048	.033	.072
M1	3	3	−269.42	0.92	0.105	T1	.035	.021	.123
M1	4	3	−269.30	1.05	0.099	P2	.033	.019	.132
M1	5	2	−269.14	1.21	0.091	(Null)			
M2	6	3	−238.54	0.00	0.270	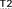	.050	.036	.064
M2	7	5	−237.32	1.22	0.147	P2 + T1+ 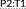	.096	.054	.087
M2	8	2	−237.16	1.38	0.135	(Null)			
M2	9	3	−237.00	1.54	0.125	P2	.029	.015	.162
M3	10	3	−257.77	0.00	0.221	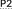	.055	.041	.052
M3	11	3	−256.51	1.25	0.118	T1	.038	.023	.109
M3	12	2	−256.04	1.73	0.093	(Null)			
M4	13	3	−248.22	0.00	0.170	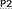	.069	.055	.029
M4	14	4	−246.28	1.94	0.065	T1 + T2	.073	.045	.082
Two‐clade analyses									
M1	15	2	−240.01	0.00	0.154	(Null)			
M1	16	3	−239.60	0.42	0.125	Clade	.028	.012	.190
M1	17	3	−239.58	0.43	0.124	T2	.028	.012	.192
M1	18	3	−239.55	0.47	0.122	P2	.028	.012	.196
M1	19	3	−239.39	0.62	0.113	T1	.025	.009	.217
M2	20	2	−212.71	0.00	0.136	(Null)			
M2	21	3	−212.51	0.20	0.123	T2	.032	.016	.165
M2	22	3	−212.17	0.54	0.104	T1	.027	.010	.205
M2	23	3	−211.96	0.75	0.094	Clade	.023	.007	.236
M2	24	3	−211.94	0.77	0.093	P2	.023	.007	.239
M3	25	3	−228.77	0.00	0.217	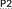	.055	.040	.065
M3	26	3	−227.76	1.01	0.131	Clade	.040	.024	.120
M3	27	2	−227.45	1.33	0.112	(Null)			
M3	28	3	−227.12	1.65	0.095	T1	.030	.014	.178
M4	29	7	−223.17	0.00	0.735	Clade + **P2** + T1 + Clade:P2 + P2:T1	.236	**.167**	.009

Note

Response variables (natural log‐transformed): M1, mean for males with SVL ≥ 45 mm; M2, mean for males defined as “adults” by primary researchers; M3, 80th percentile for males with SVL ≥ 45 mm; M4, 80th percentile for males defined as “adults” by primary researchers. Predictors: Clade, clade identity; T1, mean temperature of coldest quarter (winter temperature); T2, mean temperature of warmest quarter (summer temperature); P2, precipitation of warmest quarter (summer precipitation). Significance of individual predictors: underlined with dots, *p* < .1; underlined, *p* < .05; bold, *p* < .01; underlined bold, *p* < .001. See Section 2 for details.

### 3.2 Candidate models for female size

**Figure 2:**
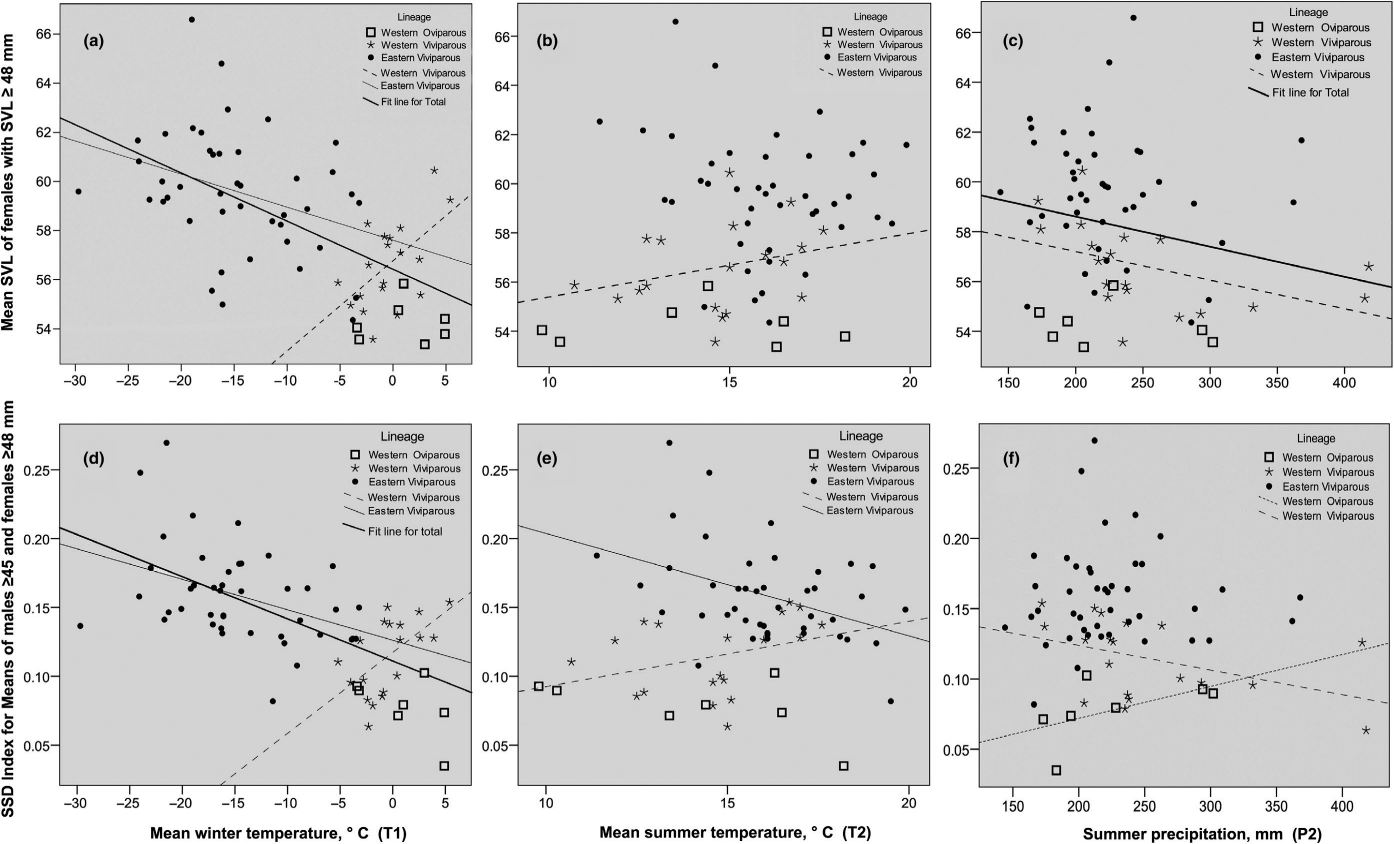
(a‐c) Female body size (snout‐vent length, SVL) and (d‐f) sexual size dimorphism (SSD) in *Zootoca vivipara* plotted against three climatic predictors. Regression lines are shown whenever the slopes differ from zero at *p* < .1

**Figure A2:**
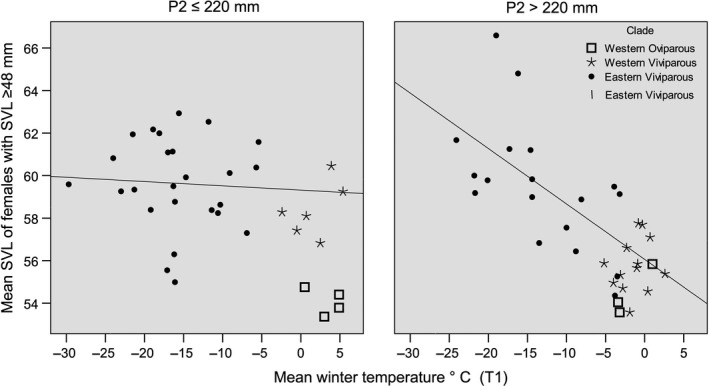
The relationship between female body size and winter temperature (T1, our proxy of seasonality) in *Zootoca vivipara* is modulated by summer precipitation (P2) resulting in a significant T1×P2 interaction (Table 3). Fit line on the right panel indicates a significant relationship (*p* < .01), the corresponding line on the left panel is given for comparison

Compared to males, body size variation in females is clearly larger (range of sample means is circa 16 mm, min–max 51–67 mm, Table A1), with much greater part of this variation being explained by our predictors (26%–49%, Table 3). In the three‐clade analyses, all five candidate models include Clade and P2 (summer precipitation); three models include T1 (winter temperature), two of them also including the T1 × P2 interaction (Table 3). In the two‐clade analyses, the top models explain a smaller proportion of the total variance than in the three‐clade analyses (26%–36% vs. 40%–49%, Table 3); they are less consistent among different metrics, and the total number of candidate models is larger (13 vs. 5). Only around half of the candidate models include Clade and P2 (Table 3). T1, which is present in 11 of 13 candidate models in the two‐clade analyses, “replaces” Clade (its collinear counterpart, see Section 2.3) as the most consistent predictor. Model coefficients of T1 were always negative, that is, female size overall increases with decreasing T1. As in the three‐clade analyses, a considerable number of candidate models (5 of 13, three of them being the top models, Table 3) include the P2 × T1 interaction. Note that the latter two predictors are also present in the only significant model for male size (Table 3). Model coefficients of P2 were consistently negative (see also Figure 2c), thus supporting our Prediction 3 (Table 1). The P2 × T1 interaction indicates that the major relationship, female size*—*T1 (Figure 2a), is modulated by covariate P2: in the eastern viviparous clade, this relationship is stronger at higher than at lower values of P2 (Appendix A8: Figure A2).

**Table 3:** AICc‐selected models (ΔAICc ≤ 2) for female size (SVL)

Metric	Model	*df*	AICc	ΔAICc	Weight	Formula	*R* ^2^	Adj *R^2^*	*p*
Three‐clade analyses									
F1	1	7	−257.11	0.00	0.496	**Clade** + 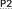 + T1 + P2:T1	.530	.492	2.7 × 10^–9^
F1	2	7	−256.21	0.89	0.318	**Clade** + T1 + Clade:T1	.524	.486	4.0 × 10^–9^
F2	3	7	−223.24	0.00	0.733	Clade + 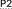 + T1 + P2:T1	.452	.409	2.7 × 10^–7^
F3	4	5	−234.45	0.00	0.403	**Clade** + P2	.425	.398	6.7 × 10^–8^
F4	5	5	−226.55	0.00	0.416	**Clade** + P2	.430	.404	5.1 × 10^–8^
Two‐clade analyses									
F1	6	5	−227.90	0.00	0.273	**Clade** + T1 + Clade:T1	.387	.355	2.7 × 10^–6^
F1	7	5	−227.52	0.38	0.227	P2 + **T1 **+ P2:T1	.383	.351	3.2 × 10^–6^
F2	8	5	−202.27	0.00	0.507	P2 + **T1 **+ P2:T1	.357	.324	1.0 × 10^–5^
F2	9	5	−200.27	2.00	0.187	Clade + T1 + Clade:T1	.336	.302	2.6 × 10^–5^
F3	10	5	−208.26	0.00	0.104	P2 + **T1 **+ 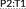	.293	.257	1.5 × 10^–4^
F3	11	4	−208.08	0.18	0.095	**Clade** + P2	.263	.239	1.2 × 10^–4^
F3	12	5	−207.51	0.75	0.072	**T1** + 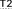 + T1:T2	.285	.248	2.1 × 10^–4^
F3	13	5	−207.02	1.24	0.056	**Clade** + T1 + Clade:T1	.279	.242	2.6 × 10^–4^
F3	14	4	−206.63	1.63	0.046	P2 + **T1**	.246	.220	2.4 × 10^–4^
F4	15	7	−204.27	0.00	0.265	P2 + **T1** + T2 + 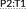 + T1:T2	.391	.337	2.9 × 10^–5^
F4	16	5	−204.00	0.27	0.231	**T1** + T2 + **T1:T2**	.337	.303	2.5 × 10^–5^
F4	17	6	−202.38	1.89	0.103	Clade + P2 + T1 + 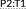	.346	.300	6.1 × 10^–5^
F4	18	4	−202.37	1.90	0.102	**Clade** + P2	.293	.269	3.7 × 10^–5^

Note

Response variables (natural log‐transformed): F1, mean for females with SVL ≥ 48 mm; F2, mean for females defined as “adults” by primary researchers; F3, 80th percentile for females with SVL ≥ 48 mm; F4, 80th percentile for females defined as “adults” by primary researchers. Other designations as in Table 2.

### 3.3 Candidate models for SSD and the shared patterns of female size and SSD variation

Sexual size dimorphism was consistently female‐biased: SSD index for means varied from 0.04 to 0.27 (Appendix A7: Table A1); that is, females were on average 4%–27% longer in SVL than males. In both the three‐clade and the two‐clade data sets, the major axis regression slope of log(male SVL) on log(female SVL) was significantly lower than 1 (Table 4). This pattern corresponds to a converse of Rensch's rule; that is, the SSD variation is primarily due to variation in female rather than male size.

**Table 4:** Major axis regression slopes of male size on female size (log‐transformed mean SVL) among populations within and across lineages of *Zootoca vivipara*

Data set	Slope estimate (95% C.I.)	Pearson correlation coefficient (*r*) between male and female SVL
All three clades, *n* = 69	0.571 (0.421–0.743)	.663***
Two viviparous clades *n* = 62	0.661 (0.490–0.863)	.684***
Western viviparous, *n* = 19	0.841 (0.422–1.554)	.649**
Eastern viviparous, *n* = 43	0.814 (0.591–1.099)	.720***

Note

The presented analyses use metric 1 as an estimator of sex‐specific adult body size (see Section 2); using other metrics results in similar patterns.

** *p* < .01,

*** *p* < .001.

**Figure A3:**
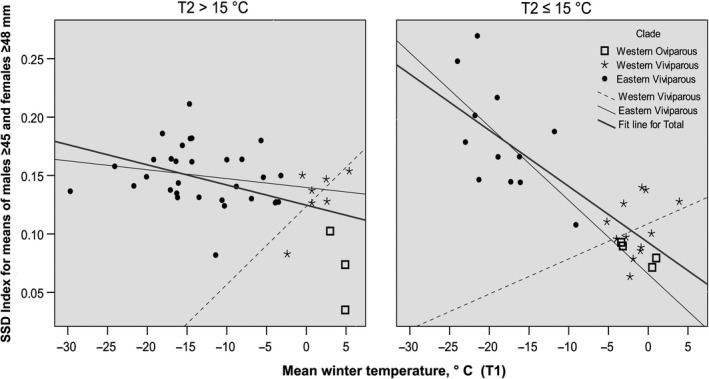
The relationship between sexual size dimorphism (SSD) and winter temperature (T1, our proxy of seasonality) in *Zootoca vivipara* is modulated by summer temperature (T2) resulting in a significant T1 × T2 interaction (Table 5). Fit lines for the western oviparous clade are not shown because of small sample sizes. Note that the opposite pattern of the SSD—Seasonality relationship in the western and eastern viviparous clades persists in both subsets of populations differing in summer temperature

The variation in SSD is even better explained by our predictors (up to 58%, Table 5) than that of absolute female size. Model selection shows a high consistency in both the three‐ and the two‐clade data sets. None of the top models for SSD includes P2, an important predictor of the female size models (see above). Another discordance with the female size models is a consistent presence of the T1 × T2 interaction, a predictor infrequently occurring in the models for female size. This interaction indicates that in the eastern viviparous clade, as well as for the whole data set, the negative SSD*—*T1 relationship is stronger at lower than at higher values of T2 (Appendix A8: Figure A3).

**Table 5:** AICc‐selected models (ΔAICc ≤ 2) for sexual size dimorphism [SSD, here (female SVL/ male SVL) ‐ 1]

Metric	Model	*df*	AICc	ΔAICc	Weight	Formula	*R* ^2^	Adj *R* ^2^	*p*
Three‐clade analyses									
D1	1	9	−287.09	0.00	0.718	Clade + T1+T2 + Clade:T1 + T1:T2	.618	.575	1.0 × 10^–10^
D2	2	9	−264.07	0.00	0.719	Clade + T1+T2 + Clade:T1 + T1:T2	.599	.553	4.0 × 10^–10^
D2	3	7	−262.19	1.88	0.281	**Clade** + 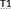 +T2 + **T1:T2**	.556	.520	5.0 × 10^–10^
D3	4	9	−236.94	0.00	0.632	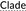 + T1+T2 + Clade:T1 + T1:T2	.468	.407	1.3 × 10^–6^
D3	5	7	−235.85	1.08	0.368	**Clade** + T1+Clade:T1	.417	.370	1.7 × 10^–6^
D4	6	9	−240.87	0.00	0.679	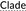 + T1+T2 + Clade:T1 + **T1:T2**	.517	.461	8.5 × 10^–8^
Two‐clade analyses									
D1	7	7	−257.76	0.00	0.312	**Clade** + **T1**+T2 + Clade:T1 + T1:T2	.515	.471	7.0 × 10^–8^
D1	8	7	−256.06	1.70	0.134	P2 + **T1**+T2 + P2:T1 + **T1:T2**	.501	.457	1.5 × 10^–7^
D2	9	7	−235.14	0.00	0.399	Clade + T1+T2 + 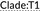 + T1:T2	.470	.422	7.8 × 10^–7^
D2	10	5	−235.01	0.13	0.375	**T1** + T2+**T1:T2**	.424	.394	4.6 × 10^–7^
D3	11	5	−215.04	0.00	0.200	**T1** + T2+**T1:T2**	.298	.262	1.2 × 10^–4^
D4	12	5	−217.87	0.00	0.454	**T1** + T2+**T1:T2**	.347	.313	1.6 × 10^–5^

Note

SSD metrics D1, D2, D3, and D4, used as response variables, are based on the corresponding metrics for sex‐specific SVL defined in Tables 2 and 3. Other designations as in Table 2.

**Figure 3:**
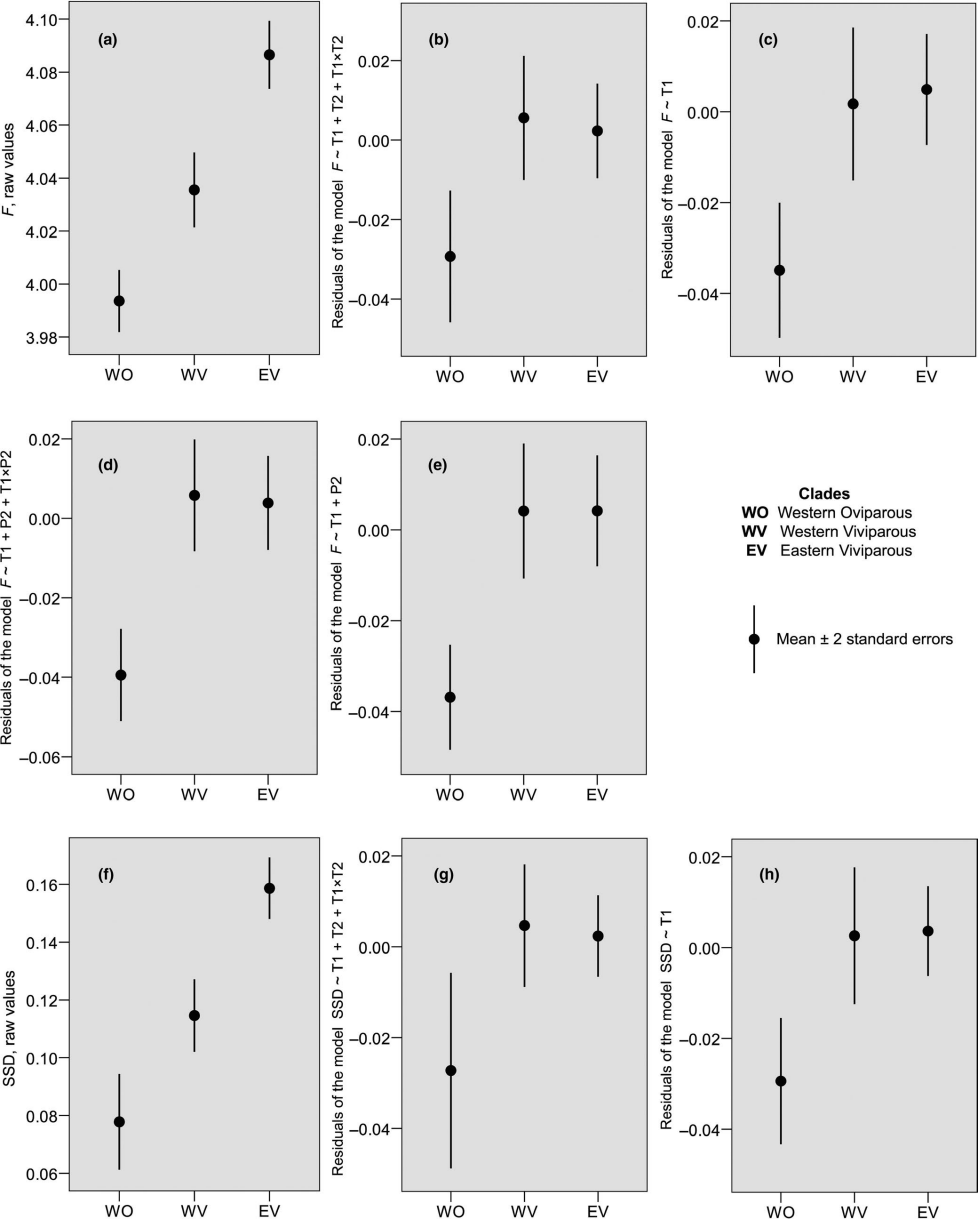
Variation in female size (*F* = LN[female size]) and sexual size dimorphism (SSD = female size/male size − 1) among three major clades of *Zootoca vivipara* based on raw values and on residuals of several models which include climatic predictors only. Presented are all models with ΔAICc ≤ 2 and two simpler models (C, H) which are useful for analytical purposes. Models are specified on the *Y* axes. The presented analyses use metric 1 as an estimator of sex‐specific adult body size (see Section 2); using other metrics results in similar patterns

The following patterns are common to the female size and the SSD variation. First, candidate models for both traits frequently include the Clade × T1 interaction (Table 3, Table 5) that reflects opposing female size*—*T1 and SSD*—*T1 relationships in the two viviparous clades (Figure 2a,d). Both correlations, a positive in the western viviparous clade and a negative in the eastern viviparous clade, are significant for several metrics of female size and SSD, the difference between the two correlation coefficients being significant for all eight metrics (Appendix A8: Table A3). The negative female size*—*T1 and SSD*—*T1 correlations for the whole data set are highly significant too (Table A3). The negative correlation corresponds to a pseudo‐Bergmann's cline (Table 1: Prediction 6), while the positive one corresponds to its converse (Table 1: Prediction 5). Second, the main effect of T1 is much stronger than that of T2: (a) while ten models include T1 but not T2, no models show the opposite pattern (Table 3, Table 5); (b) in no model, T2 exhibits a significant main effect. Thus, no support for our Predictions 1 or 2 (Table 1) was found. Third, as with female size, clade identity is a consistent predictor of SSD in the three‐clade analyses (it occurs in all six candidate models, Table 5) and becomes inconsistent in the two‐clade analyses, where four of the six candidate models include climatic predictors only (Table 5); the top models explain a smaller proportion of the total variance in the two‐clade analyses than in the three‐clade analyses (26%–47% vs. 41%–58%, Table 5). To further explore this issue, we visualized between clade differences in female size and SSD as they appear with and without controlling for climatic covariates (Figure 3). In line with Prediction 8 (Table 1), the western oviparous clade exhibits a smaller female size and less female‐biased SSD than the western and the eastern viviparous clades, whereas differences between the two viviparous clades become insignificant when controlling for climatic variables.

## 4 DISCUSSION

We investigated intraspecific divergence of adult body length in a wide‐ranging lizard across the temperate Eurasia. Using general linear models, we tested the effects of mean summer temperature, summer precipitation, seasonality, and reproductive mode/lineage identity on female size, male size, and SSD. We found a moderate effect of reproductive mode and precipitation and a strong but complex effect of seasonality. The latter differed drastically between the lineages, being also modulated by precipitation and especially by temperature. Female size and SSD varied stronger than male size, and virtually all the effects were strongly female‐biased. Below, we relate the revealed body size patterns to several evolutionary and ecological hypotheses (Table 1).

### 4.1 Allometry of SSD

Amniotes, including reptiles, tend to exhibit standard Rensch's allometry meaning that male size varies more than female size (Fairbairn, 1997). This trend is mainly expressed among species (reviewed in Fairbairn et al., 2007) but was also reported for variation among conspecific populations (e.g., Saino & De Bernardi, 1994; Garel, Solberg, Sæther, Herfindal, & Høgda, 2006; Aglar & López‐Darias, 2016), even in species with overall female‐biased SSD (Pearson et al., 2002). In contrast, SSD variation in *Z. vivipara* follows a converse of Rensch's rule. In *Z. vivipara*, male size varies not only lower but also qualitatively less regular in relation to our predictors, as compared to female size. This pattern is not solely due to a divergence between oviparous and viviparous populations (see below), as it persists in our two‐clade analyses (the western + eastern viviparous clades). Obviously, the revealed SSD allometry is also shaped by a steeper slope of the body size*—*climate relationship in females versus males, a pattern which is opposite to a prevailing trend found in a meta‐analysis of 98 animal species (Blanckenhorn et al., 2006).

### 4.2 Temperature and water availability during the active season

A strong sexual difference in the extent of body size variation, and in the percentage of this variation explained by our predictors, reduces the relevance of the hypotheses that apply equally to both sexes. This concerns the adaptive hypotheses related to heat acquisition, heat conservation, dehydration resistance, and fasting endurance (Table 1). In accordance with this reasoning, Predictions 1 and 2, which are associated with heat acquisition and heat conservation mechanisms (Table 1), received no support in our analyses: The main effect of the corresponding predictor, mean summer temperature (T2), was consistently weak. The temperature‐size rule, which also makes Prediction 2 (Table 1), thus received no support as well. These results are in line with the notion that in advanced behavioural thermoregulators, ambient temperature may not be as important as the amount of time available for thermoregulation (Adolph & Porter, 1993; Sears & Angilletta, 2004; Uller & Olsson, 2003).

Prediction 3, that is, a negative correlation of body size with summer precipitation (P2), received moderate support in this study. It is shared by the dehydration resistance hypothesis and a negative effect of precipitation on insolation, and thus directly on body growth of heliothermic organisms, such as lizards or insects (Table 1). Previous studies on lizards, including *Z. vivipara*, made on a small geographic scale in warmer regions (Díaz, Iraeta, Verdú‐Ricoy, Siliceo, & Salvador, 2012; Dunham, 1978; Lorenzon, Clobert, & Massot, 2001; Taylor, 2003) reported a positive effect of precipitation or humidity on body size (Prediction 4). The opposite pattern revealed at a large geographic scale (Figure 2c) could mean that in cooler climates, which occur on a major part of the study species range, the negative, immediate effect of precipitation on animal's activity (Table 1) exceeds the positive, delayed effect on habitat productivity (Table 1).

### 4.3 Seasonality

The strongest and most interesting pattern revealed in this study concerns the relationship of female size and SSD with mean winter temperature (T1) which is tightly correlated to seasonality and used as proxy for the length of activity season. The western viviparous clade exhibits a converse pseudo*‐*Bergmann's cline corresponding to the Adolph and Porter (1993) “null physiological model” (Table 1: Prediction 5). In contrast, a standard pseudo*‐*Bergmann's cline found in the eastern viviparous clade, as well as across the clades, corresponds to their main model (Table 1: Prediction 6) that considers shifts in the age at maturity (Adolph & Porter, 1996). Phenotypic responses predicted by Adolph and Porter (1993, 1996) models can be female‐biased due to sex‐differential plasticity of growth and maturation (Fairbairn, 2005; Cox & John‐Alder, 2007; Cox & Calsbeek, 2010; see also Table 1).

**Figure 4:**
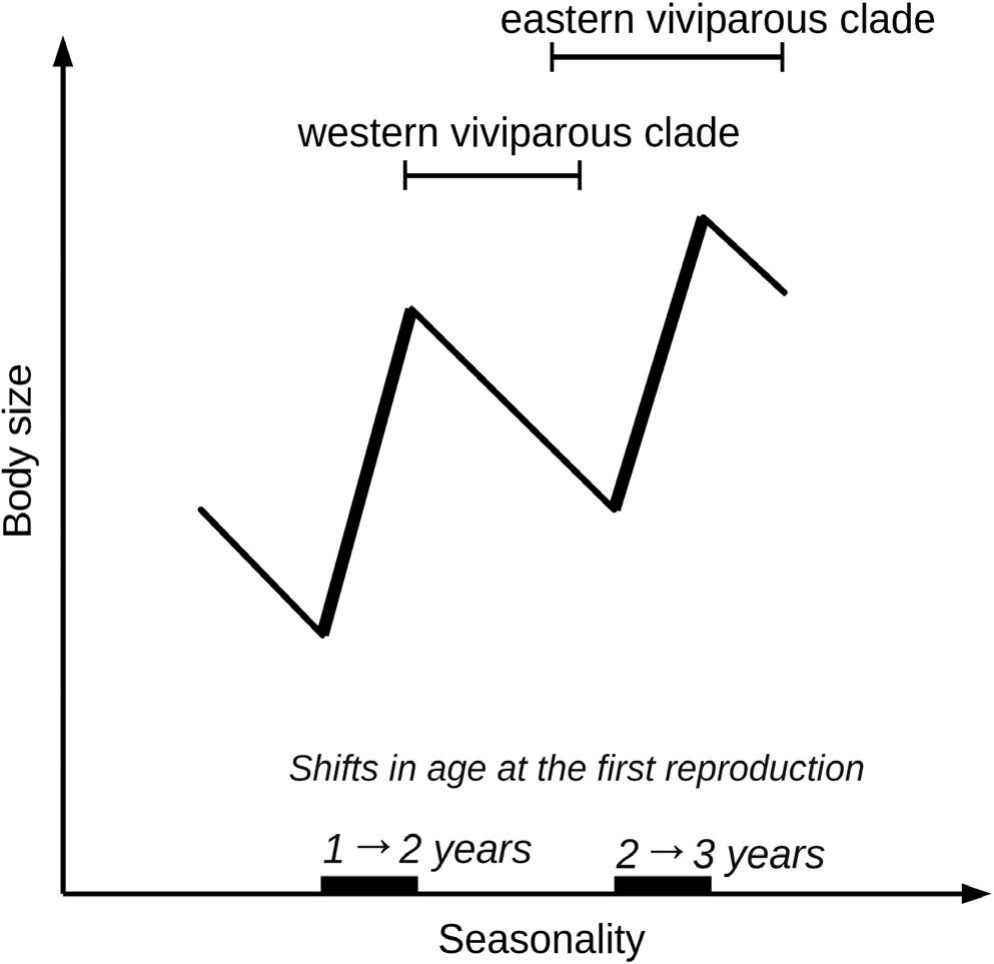
The saw‐tooth relationship between population's typical adult female size and seasonality in the lizard *Zootoca vivipara*, as hypothesized from Adolph and Porter's (1993, 1996) models. Thin line segments correspond to constant ages at the first reproduction, where the body size*—*seasonality relationship follows Adolph and Porter's (1993) null model. Thick segments indicate thresholds at which the age at the first reproduction changes abruptly resulting in a reversed body size*—*seasonality relationship (Adolph & Porter, 1996). See text for explanations

We hypothesize that geographic variation in age at maturity, specifically in females, is minor in the western viviparous clade, while pronounced in the eastern viviparous clade, at least in our data set. Available data on typical age at maturity in different populations of *Z. vivipara* appear to be in line with this hypothesis. In virtually all western viviparous populations, which were studied demographically, the modal age at maturity is 2 years (Bauwens & Verheyen, 1987; Heulin, 1985; Pilorge, 1987; Pilorge & Xavier, 1981; Strijbosch & Creemers, 1988; S. Hofmann, unpublished data). Demographic data are scarce for the eastern viviparous clade, but the modal age at maturity is expected to be generally higher and apparently more variable than in the western viviparous clade, because its geographic distribution (Figure 1) results in a strongly higher range of experienced seasonality (Figure 2a). Indeed, in the south of West Siberia the typical age at maturity was found to range from 2 to at least 3 years (Bulakhova, Kuranova, & Savelyev, 2007; Epova, Kuranova, Yartsev, & Absalyamova, 2016), being probably even higher in still more severe climates of the Middle and East Siberia. The above considerations allow us to suggest that the opposite correlations of female size (and SSD) with mean winter temperature found in the two major clades of *Z. vivipara* may reflect a saw‐tooth cline along a seasonality gradient predicted by the Adolph and Porter (1996) model (Figure 4).

A stronger effect of seasonality (as estimated by winter temperature) on female body size and particularly SSD in cooler versus warmer summer climates (T1 × T2 interaction; Tables 3 and 5; see also Appendix A8: Figure A3) is beyond our predictions. Perhaps at lower ambient temperatures, lizards use more of the potential activity period predicted by the Adolph and Porter (1993) “null” model to compensate for process limitations (sensu Congdon, 1989). In contrast, in warmer summer climates, lizards might often reduce their activity to avoid predation risk (Werner & Anholt, 1993; see also Sears, 2005), as well as the risk of overheating and dehydration, which are generally higher in such environments.

The fasting endurance hypothesis (larger individuals possess larger fat reserves and survive hibernation better than smaller individuals – Table 1), which also makes Prediction 6, cannot explain the opposing cline in the western viviparous clade and can hardly integrate a strongly female‐biased phenotypic response.

The extended “small male advantage hypothesis”, which implies a Rensch's allometry of SSD (Prediction 7b), is strongly rejected in the present study that found the opposite, converse Rensch pattern (Table 4). This converse Rensch's allometry (Prediction 7a), as well as the pseudo‐Bergmann cline in female size and SSD, corresponds well with the extended fecundity‐advantage hypothesis viewing this cline as an adaptive compensation of reduced reproduction frequency (Table 1). However, females of our study species produce a single litter per year in a wide range of climates: repeated clutches occur in considerable frequencies only in lowland oviparous populations, which constitute a tiny portion of our study samples (2 from 72); only exceptional cases of multiple clutches per season are known for viviparous populations (Horváthová et al., 2013). At the same time, no evidence for biennial or intermittent breeding so far exists for *Z. vivipara*. Furthermore, previous work (Roitberg et al., 2013) found no significant relationship of clutch size or mass with seasonality, the relationship of these reproductive traits with summer temperature being negative (Roitberg et al., 2013 applied climatic variables tightly correlated to those used in the present study; see Section 2). The above points impair a straightforward application of extended fecundity‐advantage hypothesis to the body size patterns presented here (this issue will be addressed in our future work). Also, as most other presented hypotheses, the fecundity‐advantage hypothesis does not explain the converse pseudo‐Bergmann's cline in the western viviparous clade.

Thus, the explanation of the body size*—*seasonality patterns in *Z. vivipara* based on the Adolph and Porter (1993, 1996) models is clearly more parsimonious and better compatible with the currently available evidence than the possible alternative explanations discussed above and in Appendix A9. The saw‐tooth cline, to which we relate the opposing body size*—*seasonality relationships in the western and eastern viviparous clades, is a strikingly overlooked detail of the Adolph and Porter (1996) model. Although the authors considered this nonmonotonic phenotypic response as its key prediction (Adolph & Porter, 1996: p. 272), we are unaware of any empirical or theoretic contributions regarding this issue. However, comparable saw‐tooth clines have been reported in some insects where the body size*—*seasonality relationship shows a converse pseudo‐Bergmann's pattern (as predicted by Adolph & Porter's null physiological model) when number of generations per season remains constant, but reverses it when new generations per season are added (Masaki, 1967; Mousseau, 1997).

The Adolph and Porter (1993, 1996) models obviously have potential to predict the temporal dynamic of characteristic adult body size due to ongoing climate change. Note that a marked increase in mean SVL of reproducing females found in a model *Z. vivipara* population in southern France over 1988‐2000 (Chamaillé‐Jammes et al., 2006; Le Galliard et al., 2010) corresponds to a converse pseudo‐Bergmann cline, as predicted for increasing activity season length within the same age at maturity (Adolph & Porter, 1993). A further warming can reverse this trend when a major part of yearling females would reproduce (thereby impeding their further growth) because they reach the threshold size within the “reproduction window” of their second calendar year (Adolph & Porter, 1996).

Another important point can be inferred from the revealed patterns of climatic variation. Ashton and Feldman (2003) based their comprehensive meta‐analysis of ecogeographic body size clines in reptiles on mean annual temperature. In our study, mean annual temperature is rather strongly correlated with seasonality and mean winter temperature and only weakly correlated with mean summer temperature (Appendix A3), which might mean that many of the clines reported by Ashton and Feldman (2003) are actually driven by seasonality rather than environmental temperature.

Regardless which mechanism(s) underlay the disparity of body size clines in the western and eastern viviparous clades of *Z. vivipara*, our study provides the first evidence of opposing body size*—*climate relationships in clearly intraspecific units. Ashton (2001) found opposing body size clines along seasonality gradients in two closely related allopatric species of rattle snakes, *Crotalus oreganus* and *Crotalus viridis*. Another example involves less closely related, yet congeneric iguanian lizards, *Sceloporus undulatus* and *Sceloporus graciosus* (Angilletta, Niewiarowski, et al., 2004; Sears & Angilletta, 2004). Remarkably, in all three systems the lineage exhibiting a converse pseudo‐Bergmann cline (the western viviparous clade of *Z. vivipara*, *C. oreganus*, *S. graciosus*) inhabits a western part of the respective continent (Eurasia or North America) and experiences less seasonal climates, while the form showing a standard pseudo‐Bergmann cline (the eastern viviparous clade of *Z. vivipara*, *C. viridis*, *S. undulatus*) lives in more interior parts of the continent and experiences more seasonal climates. Even though these three divergences in body size*—*climate relationships apparently differ in details, this parallelism deserves more attention, because it may reflect so far unknown factors shaping body size clines in ectotherms. One pattern of this kind has recently been revealed in North American freshwater fish: body size*—*temperature relationships, whenever significant, were consistently positive in warm‐water species, while consistently negative in cool‐/cold‐water species (Rypel, 2014).

### 4.4 Reproductive mode and lineage identity

In line with Prediction 8, populations of the western oviparous clade tend to exhibit a smaller female size and less female‐biased SSD than those of both viviparous lineages. This pattern persists when controlling for climatic variables (Figure 3) indicating that clade‐specific properties, rather than only local environment, contribute to the divergent body size phenotype of this form. Albeit phylogenetic effects cannot be fully disregarded here because in terms of ancestry the western oviparous clade is less close to the western and eastern viviparous clades than the two viviparous clades to one another (Surget‐Groba et al., 2006), a distinct reproductive mode is the most likely explanation of this divergence. In congruence, females of the eastern oviparous clade show smaller mean SVL than their viviparous counterparts collected from virtually the same site, be it the western viviparous clade (Lindtke, Mayer, & Böhme, 2010) or a relic clade the central viviparous II (Recknagel et al., 2018). Yet the differences between oviparous and viviparous populations are not clear‐cut and explain a moderate part of the intraspecific variation in female size and SSD in *Z. vivipara* (Figure 2). This is a likely reason why previous range‐wide studies on *Z. vivipara* (Horváthová et al., 2013; Roitberg et al., 2013), which had less representative data sets, found no significant effect of reproductive mode on female body size.

A problematic point of this study is evaluating the main effect of clade identity (Clade) on the body size variation among viviparous populations. Clade is collinear with T1 (Appendix A6), our proxy for seasonality, which is the strongest predictor. This collinearity arises due to a very low overlap between the western and eastern viviparous clades along the seasonality gradient (Figure 2a,d) resulting from the west–east separation of their geographic distributions (Figure 1). Further complications come from a significant Clade × T1 interaction (Tables 3 and 5). Under such conditions, the absence of clade identity in some candidate models (Tables 3 and 5), as well as the fact that differences between the two viviparous lineages in female size and SSD (Figure 3a,f) became insignificant when corrected for climatic effects (Figure 3b–e,g,h), are rather suggestive than conclusive evidence of weak or nonexistent main effect of clade identity within viviparous populations. Note that Horváthová et al. (2013) also reported a small effect of ancestry on female body size in *Z. vivipara*, even though they used a finer control for phylogenetic signal than our study.

## 5 CONCLUSION

The present study confirms a major role of seasonality (as compared to mean summer temperature and precipitation) in shaping the geographic body size variation as suggested by previous work in *Z. vivipara* (Horváthová et al., 2013; Roitberg et al., 2013). We show that the body size response to the seasonality gradient is strongly female‐biased, more complex than previously thought, and is parsimoniously interpretable as a saw‐tooth cline along a gradient of activity season lengths predicted by Adolph & Porter's models (Table 1; Figure 4). Within the western viviparous clade occurring from France to Eastern Europe, female size and SSD decrease as seasonality increases. Such a response is predicted under constant age at maturity (Adolph & Porter, 1993), an overall likely condition for this region, judging from its low seasonality and from available demographic data. Within the eastern viviparous clade (Eastern Europe to Far East), as well as across the clades, female size and SSD increase with increasing seasonality. This response is predicted under varying age at maturity (Adolph & Porter, 1996), and such variation, being confirmed by scarce empirical evidence, seems very likely, given the high and strongly variable levels of seasonality of this huge territory. Studies on life‐history and demography of *Z. vivipara* populations experiencing contrasting levels of seasonality should test whether the opposing body size clines revealed in the two widespread lineages are driven by the mechanisms underlying Adolph & Porter's models. Further, our study demonstrates that not only males (e.g., Aglar & López‐Darias, 2016) but also females can be a driver of intraspecific divergence in amniotes.

## CONFLICT OF INTEREST

The authors declare no conflict of interest.

## AUTHOR CONTRIBUTIONS

ER designed the study. All authors provided morphometric and other data from their fieldwork or museum samples; SH provided climatic data. ER conducted analyses and wrote the manuscript, to which all coauthors contributed and gave final approval for publication.

## Figures and Tables

**Figure A1:**
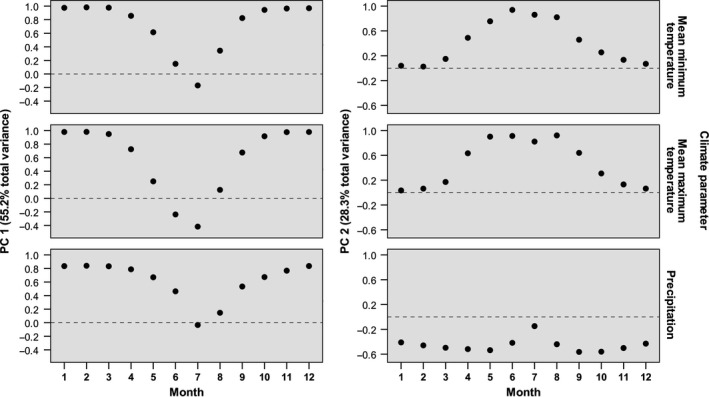
Factor loadings and percents of trace associated with the first two principal components of among‐sites variation in 36 climatic variables

## Data Availability

Original individual‐based data on body size (SVL), as well as the geographic and climatic data, are archived into the public repository Dryad https://doi.org/10.5061/dryad.r7sqv9s7v.

## APPENDIX A1

### Extraction and correction of climate data

Climatic data (monthly mean minimum and maximum temperatures, monthly mean precipitation, and several bioclimatic indices) and altitudes for the 240 study localities were obtained from the WorldClim database, version 1.4, which provides values averaged over the years 1950–2000 (Hijmans, Cameron, Parra, Jones, & Jarvis, 2005; www.worldclim.org). We used a grid cell resolution of 30 arc‐seconds and bilinear interpolation as the data extraction settings.

For a few study sites located in mountain regions the altitude value provided by WorldClim deviated substantially (200–500 m) from the value given in the original report, Google Maps or related databases (apparently due to a deviation in the reported coordinate values or local faults within the data base). An established correction for adiabatic cooling (Sears, 2005; Angilletta, Oufiero, & Leaché, 2006; Stillwell et al., 2007; Roitberg et al., 2013) would adjust the temperatures but not the precipitation. To more effectively reduce the resulting deviation in climatic values we used an original approach briefly described below. We generated an array of coordinate values (subsidiary points) around the problematic site. If the discrepancy in altitude is merely due to a small random deviation in coordinate values, as is apparently true for the vast majority of cases, then some of the subsidiary points should be closer to the “true” site than the site with the reported coordinates. Choice of the most relevant point was based on the least difference in the altitude values, the geographic proximity being also accounted for. For virtually all problematic sites of this study, at least one subsidiary point in close proximity provided a sufficiently close altitude value.

## APPENDIX A2

### Analysis of climate data

Seasonality was estimated with the coefficient of variation (CV) among monthly values of temperature or precipitations (Dobson & Wigginton, 1996; Ashton, 2001; Çağlar et al., 2014). Temperature data were transformed to Kelvin to allow calculation of the variation coefficient (Ashton, 2001).

The principal component analysis (PCA) was used to summarize the geographic variation of 36 primary climatic variables (monthly mean minimum and maximum temperatures, and monthly mean precipitations): these were reduced to a smaller set of orthogonal vectors, which include a major portion of the total variation. In our previous studies (Roitberg et al., 2013, 2015) the first two principal components, PC1‐clim and PC2‐clim, were directly used as predictors for body size and SSD in statistical models. In this study, we took advantage of the fact that PC1‐clim is tightly correlated to mean temperature of coldest quarter (T1, Worldclim code BIO11) and PC2‐clim to mean temperature of warmest quarter (T2, BIO10) (see Appendix A3). Using these two temperature indices instead of the principal components provides the following advantages. First, T1 and T2 are not confined to a given dataset and hence fully comparable among studies or among subsets of our samples. Second, their values are intuitive. Third, when using T1 and T2, we may extend our list of input variables with precipitation indices. So we included precipitation of warmest quarter (P2, BIO18) to address Predictions 3 and 4 (Table 1).

## APPENDIX A3

### Patterns of co‐variation among climatic variables across the study sites

The first axis of the principal component analysis of the climatic variables (PC1‐clim) explained 55.2% of the total variance among localities (Figure A1). PC1‐clim is strongly and positively correlated with all monthly temperature and precipitation parameters outside the warmest quarter (Figure A1); PC1‐clim is highly correlated with T1 (Spearman rank correlation coefficient, *r_s_* = .975,* p* < .001; *N* = 72) but not T2 (*r_s_* = −.016,* p* = .893). PC1‐clim is also tightly linked to temperature seasonality (*r_s_* = −.959,* p* < .001) and less strongly to precipitation seasonality (*r_s_* = −.819,* p* < .001). As expected, T1 exhibits similar correlations to these seasonality indices (−0.962 and −0.807, respectively). In contrast, the second principal component (PC2‐clim, 28.3% of the total variance, Figure A1) is strongly correlated with the monthly values of the warmer season (April–September), the loadings being consistently positive for temperatures and negative for precipitation (Figure A1). PC2‐clim is tightly related to T2 (*r_s_* = .959, *p* < .001) but not T1 (*r_s_* = .122, *p* = .307). Both PC2‐clim and T2 show no significant relations to temperature and precipitation seasonality (all *r_s_* < .21, all *p* > .09).

Mean annual temperature, the most widely used climatic index, is tightly linked to PC1‐clim, temperature seasonality, and T1 (*r_s_* = .913, −.847, and .947, respectively; all *p* < .001), being weakly correlated to PC2‐clim and T2 (all *r_s_* < .4).

## APPENDIX A4

### Considering the effects of evolutionary lineage

The phylogeographic study by Surget‐Groba et al. (2001, 2006) provides reasonably dense covering of border areas between the major clades. Thereby, virtually all our study samples could be readily assigned to particular clades based on their geographic locations (see captions to Figure 1 for two problematic samples). Surget‐Groba et al. (2001, 2006) provided phylogenetic relationships among clades (Figure 1b), but their published data gave only scarce information on the geographical distribution of haplotypes *within* the clades. Within the viviparous clades, which occupy a major part of the species range, the haplotype variation is apparently small, especially in the eastern viviparous clade (see also Takeuchi, Takeuchi, & Hikida, 2013). This variation is apparently stronger within the western oviparous clade (Milá, Surget‐Groba, Heulin, Gosá, & Fitze, 2013), but our data for this form are insufficient to address this issue. Considering the above circumstances and the fact that there are only three lineages in our study, we included clade identity as a predictor in our analyses (see above) to test for the effects of ancestry, as did Díaz et al. (2012), Roitberg et al. (2013, 2015), Ficetola et al. (2016).

## APPENDIX A5

### Considering the effects of spatial autocorrelations

To test whether the results of our GLMs are biased by spatial autocorrelation we computed spline correlograms based on Moran's *I* statistic; for this we used the ‘ncf’ package (Bjornstad, 2013) in R (R Core Team, 2018). Correlograms were computed for the residuals of the two AICc best‐fit models of each metric of each study trait. No significant autocorrelation was detected (95% confidence intervals always included zero), suggesting that spatial autocorrelation did not bias our models (Dormann et al., 2007).

## APPENDIX A6

### Considering the effects of multicollinearity

Multicollinearity between our input variables was tested by computing Generalized Variance Inflation Factor, GVIF (Fox & Monette, 1992) with the *car* package (Fox & Weisberg, 2011) in R (R Core Team, 2018). In the three‐clade analyses, GVIF amounted 3.98 for clade identity (*df* = 2), 3.39 for T1, 1.39 for T2, and 1.08 for P2. Despite their higher GVIF values, both T1 and clade identity were retained in our models as these variables have independent biological relevance. Yet, this collinearity was appreciated when processing the results of model selection (see Section 2: Data analysis).

## APPENDIX A7

### Data

**Table A1:** Summary statistics for snout‐vent length in male and female samples of *Zootoca vivipara* shaped with two different inclusion criteria (A, B). The values of sexual dimorphism index (Lovich & Gibbons, 1992) for means (SSD_x) and 80th percentiles (SSD_p80) are also given.

(A) Sample statistics for inclusion criterion 1 (adults as defined by primary researchers)																
Study sample (see Figure 1 for locations)		Clade	Males, “adults”						Females, “adults”						SSD	
ID	Sample details		*N*	Min	Max	Mean	*SD*	P80	*N*	Min	Max	Mean	*SD*	P80	SSD_x	SSD_p80
1	N Spain, W+C Cantabrians	WO	16	43.1	54.8	49.74	3.97	54.2	21	43	63	52.80	5.90	59.1	0.061	0.090
2	N Spain, E Cantabrians	WO	46	43.6	56.7	50.51	2.66	52.6	24	49	64	54.40	3.72	56.8	0.077	0.079
3	SW France, Pyrenees, Gabas	WO	54	49	61	54.06	2.72	56.0	180	49	68	58.01	4.00	62.0	0.073	0.107
4	SW France, Pyrenees, Luvie	WO	464	47	59	52.93	2.14	55.0	405	49	63	54.91	2.91	57.0	0.037	0.036
5	SW France, Pyrenees, mixed	WO	16	43	53	48.08	2.80	51.0	22	44	62	50.28	5.43	55.4	0.046	0.087
6	SW France, C Pyrenees, N slope	WO	6	43.2	55.2	48.42	4.11	52.8	26	44	60	52.68	4.86	57.8	0.088	0.095
7	NE Spain, Pyrenees, Aran Valley	WO	35	43.5	54.8	48.86	2.74	51.4	88	44	64	53.05	3.97	56.5	0.086	0.100
8	British Isles	WV	5	52	55	53.60	1.52	55.0	11	49	73	60.45	7.42	66.8	0.128	0.215
9	NW France, Paimpont	WV	144	40	58	51.02	3.16	54.0	208	45	69	58.79	4.23	63.0	0.152	0.167
10	S France, Besse	WV	58	40	59	50.19	4.12	54.0	128	45	70	56.31	5.30	61.0	0.122	0.130
11	S France, Cevennes 1	WV	34	46.9	55.5	51.09	2.29	53.0	78	49	68	58.52	3.81	61.6	0.145	0.163
12	S France, Cevennes 2	WV	166	42.3	59.7	51.15	3.52	54.0	238	44	67	54.43	4.99	59.0	0.064	0.092
13	S France, Cevennes 3	WV	50	42.6	59	48.76	3.95	52.7	130	44	65	53.64	4.52	57.1	0.100	0.083
14	Switzerland, Berner Voralps	WV	99	41	58.1	48.13	3.44	51.1	87	42	65	54.20	4.96	58.2	0.126	0.139
15	Belgium, Kalmthout	WV							182	43	60	51.48	3.46	54.0		
16	Netherland 1 (Overasselt )	WV	205	40	58	48.40	3.67	51.0	195	43	73	55.33	6.28	61.0	0.143	0.196
17	Netherland 2 (Hammert)	WV	147	41	56	47.65	3.58	51.0	148	41	65	53.56	5.52	59.0	0.124	0.157
18	W Germany	WV	29	44.9	56.6	49.42	2.85	52.2	28	41	65	51.63	6.68	58.6	0.045	0.123
19	N Germany	WV	33	43.1	60	50.28	3.65	52.9	26	44	67	55.83	6.48	62.1	0.110	0.174
20	SW Scandinavia	WV	17	48	58	54.31	2.91	57.0	17	50	66	58.28	5.32	63.1	0.073	0.107
21	E Germany	WV	11	46.1	53	50.71	2.26	52.9	26	48	65	57.04	5.10	63.2	0.125	0.195
22	E Germany, near Leipzig	WV	104	40	60	48.96	5.15	54.0	69	48	71	58.51	5.66	64.0	0.195	0.185
23	C Germany, Thüringia, Altenfeld	WV	26	42	56	49.37	3.03	52.0	19	46	60	53.17	4.20	57.0	0.077	0.096
24	N & W Austria + Bayern Alps	WV	19	44	61	52.27	4.27	55.2	13	50	65	56.60	4.59	61.2	0.083	0.109
25	S Austria + N Italy	mixed	13	45.6	57	52.15	3.16	55.0	39	47	69	58.68	5.68	63.8	0.125	0.161
26	E Austria	mixed	12	42.3	57.7	51.45	3.85	53.0	12	50	65	58.15	4.75	63.2	0.130	0.192
27	Czechia	WV	132	40.9	59.6	48.51	3.83	51.7	118	43	69	53.23	5.52	57.6	0.097	0.113
28	Slovakia	WV	45	41.5	59.8	49.23	4.13	52.3	42	45	64	53.91	5.23	59.4	0.095	0.134
29	S Serbia	WV	31	46.2	55	50.32	2.79	53.4	45	48	67	55.88	4.62	60.6	0.110	0.134
30	W Ukraine, Carpathians 1	EV	38	39.5	57	46.66	4.33	51.0	36	41	63	53.73	5.72	59.0	0.151	0.157
31	W Ukraine, Carpathians 2	EV	31	43	58.5	50.90	3.90	54.8	27	47	69	58.69	5.14	63.0	0.153	0.150
32	N Romania, Carpathians 3	EV	17	43.8	54	47.31	2.84	50.3	17	46	60	53.45	4.08	57.3	0.130	0.139
33	lowland W & C Ukraine	EV	27	43	58.2	52.43	3.22	55.0	15	49	72	59.48	6.58	66.0	0.134	0.200
34	Finnland	EV	7	44	59.3	52.80	5.71	58.2	12	48	69	59.09	5.72	64.0	0.119	0.100
35	near St Petersburg	EV	46	44.3	55.7	50.52	3.28	54.0	14	48	66	57.30	5.58	62.2	0.134	0.152
36	Novgorod R, Valday	EV	63	41	57	48.57	3.51	52.0	122	42	66	55.75	5.43	60.0	0.148	0.154
37	E Ukraine 1	EV	29	43	57	50.36	4.00	55.0	25	49	71	60.38	7.06	68.8	0.199	0.251
38	E Ukraine 2	EV	26	43	59	53.15	4.71	57.3	19	47	70	61.37	7.24	68.0	0.155	0.187
39	Moscow R	EV	24	44	54	50.06	2.61	53.0	26	51	67	58.88	4.76	64.1	0.176	0.210
40	Arkhangelsk R	EV	24	43	59.5	51.89	5.16	57.0	44	51	72	62.53	4.66	66.0	0.205	0.158
41	Mordov R	EV	32	41	57	50.19	4.54	55.0	26	43	71	57.65	6.27	63.0	0.149	0.145
42	Penza R	EV	33	40	56	51.52	3.90	55.0	27	44	70	57.19	6.98	63.4	0.110	0.153
43	Samara R	EV	28	48	60	53.96	3.71	58.0	24	51	67	58.38	5.04	65.0	0.082	0.121
44	Perm R, Chepets	EV	35	40	57	47.37	6.01	55.0	32	43	68	55.47	8.37	64.4	0.171	0.171
45	Komi R., Pechora‐Ilych nature reserve	EV	18	43.5	67.2	54.90	5.26	59.0	12	54	76	64.80	7.17	71.5	0.180	0.213
46	Perm R, Kvazhva	EV	27	42	56	49.93	3.98	54.4	24	49	66	56.83	4.49	60.0	0.138	0.103
47	Perm R, Kamenka	EV	111	42	60	49.67	3.33	52.6	141	48	72	59.00	5.41	64.0	0.188	0.217
48	W Siberia, North	EV	10	43.7	56	50.24	4.07	53.3	10	48	61	58.16	4.53	61.2	0.158	0.147
49	Yugra, SW (Konda)	EV	15	43	55	47.73	3.96	52.7	20	43	65	53.51	6.00	59.6	0.121	0.130
50	Yugra, Severnyi	EV	9	44.7	52.3	48.34	2.50	50.9	14	45	70	60.73	7.00	66.4	0.256	0.306
51	Yugra, Ob River, W	EV	36	42	57	49.41	3.66	53.5	37	43	73	56.69	7.48	64.0	0.147	0.195
52	Yugra, Ob River, mid & E	EV	41	48	57	52.13	2.23	54.1	38	47	71	59.11	5.86	64.6	0.134	0.194
53	Yugra, Sibirskiye Uvaly	EV	40	45	57	50.28	3.17	53.0	27	45	74	57.75	6.93	63.9	0.149	0.205
54	Krasnoyarsk R	EV	20	42	55	47.60	3.82	50.8	19	49	68	60.82	5.11	65.0	0.278	0.280
55	Tomsk R, S taiga	EV	13	48.9	60.3	52.98	2.71	54.3	85	54	72	62.25	4.11	65.7	0.175	0.210
56	Tomsk R, Kireyevskoye	EV	15	47.2	59	53.65	3.14	56.9	30	46	75	61.69	5.48	65.3	0.150	0.148
57	Tomsk R, Timiryazevo	EV	173	43.8	57.8	51.81	2.91	54.2	319	46	71	59.18	4.20	62.7	0.142	0.156
58	Tomsk R, Kuzovlevo	EV	28	46	59.7	53.03	2.90	56.0	88	51	70	59.60	3.88	63.0	0.124	0.125
59	Tomsk R, Anikino	EV	30	44.4	59	49.61	3.13	51.6	32	49	72	57.37	5.86	62.5	0.156	0.210
60	Kuz. Alatau, N	EV	7	47.6	57.7	52.47	3.60	56.1	19	52	68	61.09	3.74	64.2	0.164	0.145
61	Kuz. Alatau, W	EV	17	45	61	53.63	4.05	56.1	27	53	76	63.36	5.38	68.0	0.181	0.212
62	Kuz. Alatau, E	EV	13	50.8	60	55.33	2.85	58.1	23	60	74	67.00	4.64	72.4	0.211	0.245
63	Kemerovo R, Shoria	EV	28	50.6	58.5	54.76	1.94	56.3	17	56	71	62.69	4.28	67.2	0.145	0.193
64	N Altai	EV	12	44.6	53.1	49.07	2.41	51.2	26	47	72	59.82	6.19	64.5	0.219	0.261
65	NE Altai, lowland	EV	45	45	59	51.79	2.89	54.0	41	46	75	60.83	6.03	66.0	0.175	0.222
66	NE Altai, highland	EV	21	43	59	52.38	4.72	57.0	64	45	76	60.88	7.05	67.0	0.162	0.175
67	SW Altai, Markakol	EV	20	43	52	47.81	2.28	50.1	40	46	68	54.14	5.04	58.8	0.132	0.173
68	area around Baikal Sea	EV	20	43	55.1	48.54	4.01	52.8	26	49	70	60.00	6.23	65.3	0.236	0.237
69	SW Yakutiya	EV	8	42	58	50.08	5.21	54.6	27	49	67	59.91	4.57	64.4	0.196	0.179
70	NE China, Sinvu	EV	42	45.8	57.3	51.86	3.13	54.7	97	46	72	58.69	5.44	63.6	0.132	0.162
71	Amur R	EV	26	47.6	59.6	53.26	2.94	55.4	22	57	68	61.67	2.63	64.0	0.158	0.155
72	S Sakhalin & N Hokkaido	EV	35	43	55	48.80	2.85	50.4	48	47	69	57.33	5.07	61.9	0.175	0.228

(B) Sample statistics for inclusion criterion 2 (males with SVL ≥ 45 mm, females with SVL ≥ 48 mm)																
Study sample (see Figure 1 for locations)		Clade	Males						Females						SSD	
ID	Sample details		*N*	Min	Max	Mean	*SD*	P80	*N*	Min	Max	Mean	*SD*	P80	SSD_x	SSD_p80
1	N Spain, W+C Cantabrians	WO	13	45.7	54.8	51.11	2.96	54.3	17	49	63	54.76	4.59	61.1	0.071	0.124
2	N Spain, E Cantabrians	WO	45	45.5	56.7	50.67	2.47	53.0	24	49	64	54.40	3.72	56.8	0.074	0.071
3	SW France, Pyrenees, Gabas	WO	160	45	61	51.73	3.89	55.0	412	48	68	55.84	4.55	60.0	0.079	0.091
4	SW France, Pyrenees, Luvie	WO	952	45	59	51.97	3.10	55.0	826	48	63	53.79	3.42	57.0	0.035	0.036
5	SW France, Pyrenees, mixed	WO	15	45	53	48.41	2.54	51.2	14	48	62	53.37	4.37	57.0	0.102	0.112
6	NE Spain, C Pyrenees, N slope	WO	5	45.2	55.2	49.46	3.60	54.0	22	49	60	54.05	3.92	58.4	0.093	0.082
7	NE Spain, Pyrenees, Aran Valley	WO	33	45.1	54.8	49.16	2.50	51.6	82	48	64	53.57	3.57	56.5	0.090	0.097
8	British Isles	WV	5	52	55	53.60	1.52	55.0	11	49	73	60.45	7.42	66.8	0.128	0.215
9	NW France, Paimpont	WV	335	45	58	51.35	3.17	54.0	594	48	69	59.25	4.92	63.0	0.154	0.167
10	S France, Besse	WV	212	45	59	50.70	3.58	54.0	321	48	70	57.69	5.50	63.0	0.138	0.167
11	S France, Cevennes 1	WV	222	45	55.5	50.68	3.35	54.0	260	48	68	57.76	6.04	63.0	0.140	0.167
12	S France, Cevennes 2	WV	571	45	59.7	51.31	3.56	55.0	683	48	67	55.85	4.32	60.0	0.088	0.091
13	S France, Cevennes 3	WV	94	45	59	51.27	3.33	55.0	201	48	65	55.66	4.28	59.0	0.086	0.073
14	Switzerland, Berner Voralps	WV	82	45	58.1	49.14	2.83	52.0	77	48	65	55.33	4.03	58.3	0.126	0.120
15	Belgium, Kalmthout	WV							157	48	60	52.39	2.75	54.4		
16	Netherland 1 (Overasselt )	WV	172	45	58	49.55	2.74	51.0	169	48	73	56.83	5.31	61.0	0.147	0.196
17	Netherland 2 (Hammert)	WV	112	45	56	49.10	2.77	52.0	121	48	65	55.38	4.30	60.0	0.128	0.154
18	W Germany	WV	28	45.9	56.6	49.58	2.77	52.2	20	48	65	54.56	5.37	60.0	0.100	0.150
19	N Germany	WV	31	45	60	50.69	3.36	53.1	23	48	67	57.10	5.71	62.2	0.126	0.172
20	SW Scandinavia	WV	18	45.5	58	53.82	3.50	57.0	17	50	66	58.28	5.32	63.1	0.083	0.107
21	E Germany	WV	13	45.1	53	49.92	2.83	52.8	25	49	65	57.42	4.81	63.6	0.150	0.205
22	E Germany, near Leipzig	WV	80	45	60	51.09	3.81	55.0	73	48	71	58.10	5.81	63.2	0.137	0.149
23	C Germany, Thüringia, Altenfeld	WV	25	45.5	56	49.66	2.69	52.0	18	49	60	53.57	3.93	57.3	0.079	0.102
24	N & W Austria + Bayern Alps	WV	17	47.8	61	53.22	3.40	55.6	13	50	65	56.60	4.59	61.2	0.064	0.101
25	S Austria + N Italy	mixed	13	45.6	57	52.15	3.16	55.0	36	50	69	59.64	4.76	63.9	0.144	0.163
26	E Austria	mixed	11	46	57.7	52.28	2.68	53.0	12	50	65	58.15	4.75	63.2	0.112	0.192
27	Czechia	WV	105	45.2	59.6	49.85	3.02	52.3	99	48	69	54.70	4.71	58.0	0.097	0.109
28	Slovakia	WV	39	45.5	59.8	50.16	3.59	53.4	37	48	64	54.96	4.64	59.8	0.096	0.121
29	S Serbia	WV	31	46.2	55	50.32	2.79	53.4	45	48	67	55.88	4.62	60.6	0.110	0.134
30	W Ukraine, Carpathians 1	EV	25	45	57	49.02	3.29	52.8	31	48	63	55.26	4.41	59.0	0.127	0.117
31	W Ukraine, Carpathians 2	EV	29	45	58.5	51.42	3.47	55.0	26	50	69	59.13	4.66	63.0	0.150	0.145
32	N Romania, Carpathians 3	EV	13	45	54	48.22	2.62	50.7	15	49	60	54.36	3.38	58.1	0.127	0.147
33	lowland W & C Ukraine	EV	26	48	58.2	52.79	2.66	55.0	15	49	72	59.48	6.58	66.0	0.127	0.200
34	Finnland	EV	6	46.4	59.3	54.27	4.59	58.5	11	54	69	60.12	4.70	64.6	0.108	0.104
35	near St Petersburg	EV	45	45.2	55.7	50.70	3.10	54.0	14	48	66	57.30	5.58	62.2	0.130	0.152
36	Novgorod R, Valday	EV	54	45	57	49.48	2.89	52.0	115	48	66	56.44	4.76	60.0	0.141	0.154
37	E Ukraine 1	EV	26	45.5	57	51.17	3.36	55.0	25	49	71	60.38	7.06	68.8	0.180	0.251
38	E Ukraine 2	EV	25	45.5	59	53.62	4.19	57.4	19	50	70	61.58	6.85	68.0	0.148	0.185
39	Moscow R	EV	22	47	54	50.59	1.99	53.0	26	51	67	58.88	4.76	64.1	0.164	0.210
40	Arkhangelsk R	EV	22	45.5	59.5	52.65	4.67	57.4	44	51	72	62.53	4.66	66.0	0.188	0.150
41	Mordov R	EV	27	45	57	51.59	3.35	55.0	25	50	71	58.24	5.62	63.0	0.129	0.145
42	Penza R	EV	31	46	56	52.16	2.99	55.0	24	50	70	58.63	5.96	64.0	0.124	0.164
43	Samara R	EV	28	48	60	53.96	3.71	58.0	24	51	67	58.38	5.04	65.0	0.082	0.121
44	Perm R, Chepets	EV	20	45	57	51.50	4.58	56.0	23	48	68	59.83	5.22	65.0	0.162	0.161
45	Komi R., Pechora‐Ilych nature reserve	EV	17	48.4	67.2	55.57	4.56	58.9	12	54	76	64.80	7.17	71.6	0.166	0.216
46	Perm R, Kvazhva	EV	26	45	56	50.23	3.72	54.6	24	49	66	56.83	4.49	60.0	0.131	0.099
47	Perm R, Kamenka	EV	147	45	60	49.91	3.05	53.0	163	48	72	58.99	5.39	64.0	0.182	0.208
48	W Siberia, North	EV	8	47.4	56	51.76	2.82	53.8	9	54	61	59.34	2.70	61.2	0.146	0.137
49	Yugra, SW (Konda)	EV	12	45	55	48.83	3.64	53.6	16	49	65	55.55	4.74	60.5	0.138	0.128
50	Yugra, Severnyi	EV	8	46.1	52.3	48.79	2.24	51.1	13	49	70	61.94	5.55	66.9	0.270	0.307
51	Yugra, Ob River, W	EV	32	45	57	50.18	3.10	53.9	32	48	73	58.39	6.52	64.4	0.164	0.194
52	Yugra, Ob River, mid & E	EV	42	47.9	57	52.03	2.29	54.0	36	50	71	59.78	5.23	64.7	0.149	0.198
53	Yugra, Sibirskiye Uvaly	EV	40	45	57	50.28	3.17	53.0	24	51	74	59.26	5.72	65.0	0.179	0.226
54	Krasnoyarsk R	EV	16	45	55	48.74	3.36	52.5	19	49	68	60.82	5.11	65.0	0.248	0.238
55	Tomsk R, S taiga	EV	16	48.3	60.3	52.27	2.88	53.8	87	49	72	61.99	4.43	65.6	0.186	0.219
56	Tomsk R, Kireyevskoye	EV	19	47	59	52.60	3.64	56.5	33	50	75	61.13	5.60	65.1	0.162	0.152
57	Tomsk R, Timiryazevo	EV	196	45.2	57.8	51.39	2.98	54.0	339	48	71	58.77	4.45	62.3	0.144	0.154
58	Tomsk R, Kuzovlevo	EV	31	46	59.7	52.43	3.32	55.5	89	51	70	59.50	3.97	63.0	0.135	0.134
59	Tomsk R, Anikino	EV	30	45.3	59	49.77	2.97	51.6	37	48	72	56.30	6.10	61.3	0.131	0.188
60	Kuz. Alatau, N	EV	7	47.6	57.7	52.47	3.60	56.1	19	52	68	61.09	3.74	64.2	0.164	0.145
61	Kuz. Alatau, W	EV	18	45	61	53.52	3.96	55.8	28	51	76	62.93	5.74	68.0	0.176	0.219
62	Kuz. Alatau, E	EV	14	46.9	60	54.73	3.54	58.1	24	57	74	66.59	4.96	72.4	0.217	0.246
63	Kemerovo R, Shoria	EV	33	45.1	58.5	53.51	3.54	56.0	21	54	71	61.25	4.91	66.1	0.145	0.180
64	N Altai	EV	11	46	53.1	49.47	2.05	51.4	26	50	72	59.92	5.98	64.5	0.211	0.254
65	NE Altai, lowland	EV	45	45	59	51.79	2.89	54.0	40	51	75	61.20	5.62	66.0	0.182	0.222
66	NE Altai, highland	EV	19	45	59	53.32	3.87	57.0	59	49	76	62.17	5.67	67.0	0.166	0.175
67	SW Altai, Markakol	EV	19	45	52	48.06	2.03	50.3	36	48	68	54.99	4.58	59.0	0.144	0.173
68	area around Baikal Sea	EV	16	45.2	55.1	49.94	3.19	53.3	26	49	70	60.00	6.23	65.3	0.201	0.225
69	SW Yakutiya	EV	6	48	58	52.43	3.31	56.3	28	49	67	59.59	4.79	64.2	0.137	0.140
70	NE China, Sinvu	EV	42	45.8	57.3	51.86	3.13	54.8	93	50	72	59.18	4.99	63.5	0.141	0.159
71	Amur R	EV	26	47.6	59.6	53.26	2.94	55.4	22	57	68	61.67	2.63	64.0	0.158	0.155
72	S Sakhalin & N Hokkaido	EV	31	45	55	49.46	2.27	50.8	47	49	69	57.55	4.89	61.9	0.164	0.219

Data sources: 1, 2: O. A. Arribas; 3, 4: Osenegg, 1995; 5: A. Clasen, E. S. Roitberg; 6, 7: O. A. Arribas; 8: Smith, 2006; Simms, 1969; 9: Heulin, 1985; 10: Pilorge & Xavier, 1981; 11–13: Pilorge, 1987; Khodadoost, Pilorge, & Ortega, 1987; 14: Cavin, 1993; 15: Bauwens & Verheyen, 1987; 16–17: H. Strijbosch et al.; 18–20: E. S. Roitberg, A. Clasen; 21: E. S. Roitberg; 22: S. Hofmann; 23: E. S. Roitberg; 24–26: E. S. Roitberg, A. Clasen; 27–28: M. Fokt; 29: K. Ljubisavljević; 30: E. S. Roitberg; 31: V. F. Orlova, O. I. Zinenko; 32: V. F. Orlova; 33: O. I. Zinenko, V. F. Orlova, E. S. Roitberg; 34: A. Clasen, E. S. Roitberg; 35: E. S. Roitberg; 36: O. A. Leontyeva et al.; 37: O. I. Zinenko, E. S. Roitberg; 38: O. I. Zinenko; 39–40: V. F. Orlova; 41–44: G. V. Eplanova; 45: Anufriev & Bobretsov, 1996; 46: G. V. Eplanova; 47: V. F. Orlova; 48: R. R. Shamgunova, V. F. Orlova; 49–53: R. R. Shamgunova et al.; 54: V. F. Orlova, I. V. Doronin; 55: V. N. Kuranova; 56: V. N. Kuranova; 57: N. A. Bulakhova, V. N. Kuranova; 58: V. N. Kuranova, N. A. Bulakhova; 59: N. A. Bulakhova, V. N. Kuranova; 60: V. N. Kuranova, N. A. Bulakhova; 61: V. N. Kuranova; 62: N. A. Bulakhova, V. N. Kuranova; 63: N. A. Bulakhova; 64: N. A. Bulakhova, V. F. Orlova; 65: V. A. Yakovlev, N. A. Bulakhova; 66: V. A. Yakovlev; 67: V. F. Orlova, E. S. Roitberg; 68: V. F. Orlova; 69: V. F. Orlova, N. A. Bulakhova; 70: Liu, Zhao, Liu, Dong, & Chen, 2008; 71: I. V. Tarasov; 72: V. F. Orlova, I. V. Doronin.

Abbreviations: P80, the 80th percentile; SD, standard deviation.

Geographic abbreviations: N, northern; S, southern; W, western; E, eastern; NW, northwestern; NE, northeastern; SW, southwestern; SE, southeastern; C, central. For Russia, from which a majority of samples comes, the country is not specified but the geographic or administrative region (R) is given. Bayern Alps (part of Sample 24) are in S Germany; Hokkaido (part of Sample 72) is in N Japan.

**Table A2:** Geographic and climatic characteristics of study samples

ID	latitude	longitude	altitude	T1	T2	P2	PC1‐clim	PC2‐clim	S‐t	S‐p	T‐a
1	42.9335	−6.0330	1622	0.5	13.4	173	1.254	−1.161	1.920	30.004	6.5
2	43.1500	−3.4330	762	4.9	16.5	194	1.745	0.171	1.660	23.640	10.4
3	42.9000	−0.4167	1165	1.0	14.4	228	1.236	−0.578	1.966	13.932	7.5
4	43.0933	−0.3792	373	4.9	18.2	183	1.632	1.134	1.905	15.319	11.6
5	42.9967	−0.3980	769	3.0	16.3	206	1.436	0.273	1.934	14.199	9.6
6	42.9244	0.1054	2069	−3.4	9.8	294	0.965	−2.898	1.979	12.692	2.8
7	42.7610	0.9055	2103	−3.2	10.3	302	0.987	−2.746	2.020	12.764	3.1
8	52.9564	−3.2919	66	3.9	15.0	205	1.414	−0.087	1.615	21.225	9.3
9	48.0833	−2.3000	82	5.4	16.7	172	1.500	0.877	1.630	22.292	11.1
10	45.5000	2.9100	1212	−0.3	13.1	263	0.855	−0.846	1.991	19.642	6.2
11	44.4500	3.7200	1405	−0.8	12.7	236	0.829	−1.062	2.013	14.397	5.7
12	44.3842	3.8778	1416	−0.9	12.7	237	0.848	−1.108	2.026	13.779	5.7
13	44.3850	3.8950	1443	−1.0	12.5	238	0.843	−1.210	2.016	13.607	5.5
14	46.5470	7.7500	1443	−3.1	11.9	415	1.189	−2.221	2.227	12.512	4.4
15	51.4167	4.4167	17	3.0	16.7	202	1.248	0.784	1.999	13.988	9.9
16	51.7780	5.8130	10	2.5	16.5	217	1.132	0.764	2.038	13.777	9.6
17	51.5210	6.1630	21	2.6	17.0	224	1.172	0.931	2.086	13.777	9.9
18	50.4640	6.4963	464	0.4	14.8	277	1.162	−0.264	2.123	14.492	7.7
19	54.0828	9.8084	23	0.7	16.0	226	0.897	0.494	2.234	19.880	8.3
20	58.2381	13.0109	144	−2.4	15.1	204	0.416	0.158	2.561	26.204	6.1
21	51.2266	13.8346	221	−0.5	17.0	212	0.654	1.087	2.534	26.783	8.3
22	51.3670	12.2333	99	0.7	17.6	174	0.638	1.479	2.436	26.927	9.1
23	50.5646	10.9606	632	−1.9	14.6	235	0.619	0.006	2.415	16.440	6.5
24	47.6362	11.8636	860	−2.3	15.0	418	0.963	−0.486	2.510	34.499	6.7
25	46.6485	12.4331	1585	−4.3	11.6	388	0.594	−1.735	2.361	30.330	3.7
26	47.8644	16.3059	431	−1.3	17.4	271	0.736	0.954	2.687	33.279	8.2
27	49.4868	13.9873	679	−2.8	14.9	293	0.660	−0.105	2.570	25.051	6.3
28	48.9893	20.1442	778	−4.0	14.6	332	0.521	−0.223	2.714	39.236	5.6
29	42.5942	21.7525	1918	−5.2	10.7	223	0.438	−1.734	2.365	15.354	2.9
30	48.6717	23.5617	642	−3.5	15.7	299	0.604	0.288	2.791	34.922	6.6
31	48.3131	23.9084	555	−3.2	16.4	288	0.636	0.614	2.840	36.378	7.1
32	47.5217	25.5667	683	−3.8	16.1	286	0.428	0.670	2.899	51.425	6.7
33	50.0142	27.9598	215	−3.9	18.3	250	0.492	1.369	3.242	38.580	7.6
34	63.4077	26.6587	124	−9.1	14.2	199	−0.344	−0.359	3.413	31.188	2.2
35	60.2225	29.6822	57	−6.9	16.1	217	0.074	0.257	3.359	32.001	4.5
36	57.9913	33.3749	209	−8.8	15.5	238	−0.083	0.009	3.592	31.211	3.4
37	50.7781	34.6514	135	−5.7	19.0	198	0.321	1.588	3.627	24.227	6.9
38	49.8183	36.5617	102	−5.4	19.9	169	0.359	1.984	3.709	21.152	7.6
39	55.3489	37.7662	153	−8.1	17.4	237	0.090	0.806	3.732	31.452	4.9
40	65.9538	42.7954	61	−11.8	11.4	166	−0.763	−1.181	3.430	30.372	−0.3
41	54.8350	45.3370	181	−10.6	18.1	193	−0.239	1.077	4.249	32.212	3.9
42	52.7420	45.2280	207	−10.3	19.1	175	−0.150	1.500	4.357	26.726	4.7
43	53.6716	50.5661	69	−11.4	19.5	166	−0.164	1.642	4.589	24.990	4.5
44	60.4258	55.6450	122	−14.4	15.8	222	−0.556	−0.147	4.429	29.933	.8
45	61.8216	56.8420	137	−16.2	14.6	225	−0.773	−0.576	4.544	30.432	−0.6
46	58.3830	56.4000	158	−13.5	16.1	223	−0.477	0.173	4.364	33.962	1.7
47	57.9990	57.7780	262	−14.4	15.6	243	−0.557	−0.098	4.439	35.828	.9
48	64.3000	70.4332	26	−21.3	13.2	196	−1.523	−1.094	5.197	39.763	−4.3
49	60.4417	64.2583	53	−17.1	15.9	214	−0.968	0.124	4.852	49.289	−0.3
50	62.9170	72.2330	100	−21.5	13.4	212	−1.494	−1.083	5.263	41.347	−4.3
51	61.0278	70.1167	50	−19.2	15.5	220	−1.145	−0.223	5.158	44.841	−1.7
52	60.9334	74.7542	34	−20.1	15.2	224	−1.270	−0.343	5.282	47.351	−2.4
53	62.8333	81.4167	190	−23.0	13.4	208	−1.656	−1.240	5.482	39.912	−5.2
54	60.9859	91.5822	281	−24.0	14.5	202	−1.508	−0.861	5.779	35.239	−4.5
55	57.6814	84.1448	96	−18.1	16.3	191	−1.059	0.240	5.093	44.284	−0.6
56	56.3670	84.0830	77	−16.4	17.2	193	−0.856	0.639	4.996	41.859	.7
57	56.4922	84.9036	73	−16.1	17.3	201	−0.790	0.609	4.959	39.421	.9
58	56.5667	85.0000	100	−16.3	17.1	204	−0.805	0.511	4.953	39.080	.7
59	56.3833	85.0164	129	−16.2	17.1	207	−0.791	0.484	4.931	38.797	.7
60	55.5003	88.0593	453	−17.0	16.0	214	−0.988	0.210	4.896	50.832	−0.2
61	54.3510	87.5900	303	−15.6	17.5	209	−0.789	0.818	4.890	53.108	1.4
62	54.1798	89.2872	1073	−19.0	13.5	243	−1.258	−0.704	4.861	57.087	−2.4
63	52.9050	87.9145	951	−17.3	15.0	246	−1.007	−0.173	4.810	58.955	−0.8
64	51.6019	85.6532	795	−14.7	16.2	220	−0.766	0.400	4.601	54.824	1.1
65	51.8225	87.3174	455	−14.6	18.4	248	−0.606	1.148	4.865	70.630	2.6
66	51.0154	88.6153	1727	−18.9	12.6	167	−1.519	−0.516	4.719	72.760	−2.7
67	48.7162	86.0012	1544	−16.1	14.3	164	−1.065	0.090	4.564	53.768	−0.4
68	53.0241	106.9817	815	−21.8	14.4	262	−1.517	−0.301	5.420	84.292	−3.0
69	60.3833	120.4667	202	−29.7	16.0	144	−2.212	0.053	6.979	58.526	−6.2
70	49.6553	127.5695	305	−21.7	17.9	362	−1.140	0.694	5.906	104.267	−0.7
71	49.3375	130.3253	185	−24.1	18.7	368	−1.079	0.832	6.387	94.214	−0.9
72	46.6959	142.4901	55	−10.0	15.3	309	0.330	−0.579	3.715	32.779	3.1

See Figure 1 and Appendix A7 (Table A1) for details. S‐t, temperature seasonality; S‐p, precipitation seasonality; T‐a, mean annual temperature. See Appendix A3 for other abbreviations and Methods for data extraction details.

## APPENDIX A8

### Results

**Table A3:** Spearman rank correlations (*r_s_*) between four different metrics of male size (M1–M4), female size (F1–F4) and SSD (D1–D4) and three climatic variables (T1, T2, and P2)

Clade	statistic	*n*	D1‐T1	D2‐T1	D3‐T1	D4‐T1	F1‐T1	F2‐T1	F3‐T1	F4‐T1	M1‐T1	M2‐T1	M3‐T1	M4‐T1
Western Oviparous	*r*_s_	7	−.468	−.685	−.450	−.739	−.036	.414	−.245	−.234	.378	.450	.136	.090
	*p*		.289	.090	.310	.058	.939	.355	.596	.613	.403	.310	.771	.848
Western Viviparous	*r*_s_	19	.642	.497	.663	.591	.490	.332	.507	.464	.084	.029	−.070	.054
	*p*		.003	.031	.002	.008	.033	.165	.027	.046	.732	.906	.775	.825
Eastern Viviparous	*r*_s_	43	−.414	−.254	−.237	−.253	−.377	−.345	−.219	−.222	−.069	−.101	−.012	.026
	*p*		.006	.100	.126	.102	.013	.023	.158	.153	.659	.517	.940	.869
All three clades	*r*_s_	69	−.639	−.609	−.444	−.476	−.627	−.573	−.546	−.562	−.189	−.128	−.180	−.198
	*p*		.000	.000	.000	.000	.000	.000	.000	.000	.119	.295	.138	.102
WV versus EV (*z*)	*z*		4.063	2.721	3.515	3.170	3.153	2.383	2.641	2.462				
WV versus EV (*p*)	*p*		.0000	.003	.000	.001	.002	.009	.007	.004				

Clade	Statistic	*n*	D1‐T2	D2‐T2	D3‐T2	D4‐T2	F1‐T2	F2‐T2	F3‐T2	F4‐T2	M1‐T2	M2‐T2	M3‐T2	M4‐T2
Western Oviparous	*r*_s_	7	−.500	−.750	−.464	−.750	−.071	.429	−.216	−.214	.429	.464	.198	.143
	*p*		.253	.052	.294	.052	.879	.337	.641	.645	.337	.294	.670	.760
Western Viviparous	*r*_s_	19	.417	.332	.470	.551	.395	.296	.506	.587	.043	−.008	−.065	.055
	*p*		.075	.165	.042	.014	.094	.218	.027	.008	.861	.974	.793	.824
Eastern Viviparous	*r*_s_	43	−.357	−.255	−.103	−.169	−.146	−.015	−.046	.017	.183	.244	.144	.196
	*p*		.019	.099	.512	.279	.350	.924	.772	.914	.241	.115	.357	.209
All three clades	*r*_s_	69	.100	.145	.193	.195	.171	.236	.202	.258	.219	.226	.135	.217
	*p*		.413	.236	.113	.108	.160	.051	.096	.032	.071	.062	.270	.074

Clade	Statistic	*n*	D1‐P2	D2‐P2	D3‐P2	D4‐P2	F1‐P2	F2‐P2	F3‐P2	F4‐P2	M1‐P2	M2‐P2	M3‐P2	M4‐P2
Western Oviparous	*r*_s_	7	.714	.714	.000	.679	−.286	−.107	−.396	−.214	−.536	−.321	−.378	−.321
	*p*		.071	.071	1.000	.094	.535	.819	.379	.645	.215	.482	.403	.482
Western Viviparous	*r*_s_	19	−.504	−.495	−.480	−.514	−.682	−.582	−.642	−.658	−.289	−.267	−.149	−.301
	*p*		.028	.031	.038	.024	.001	.009	.003	.002	.229	.270	.542	.211
Eastern Viviparous	*r*_s_	43	.065	.046	.170	.180	−.124	−.122	−.164	−.140	−.211	−.160	−.278	−.262
	*p*		.679	.769	.276	.248	.428	.436	.294	.371	.175	.304	.071	.089
All three clades	*r*_s_	69	−.113	−.132	−.068	−.058	−.263	−.268	−.282	−.277	−.281	−.228	−.286	−.310
	*p*		.355	.280	.580	.637	.029	.026	.019	.021	.019	.059	.017	.010

For correlations of female size and SSD with T1, significance of the differences between the two viviparous clades is also indicated. All designations of our response and explanatory variables as in Tables 1, 2, 3.

## APPENDIX A9

### Additional hypotheses to explain body‐size—seasonality relationships in common lizards

Body size at maturity is usually tightly related to overall adult size (Shine, 1990; Adolph & Porter, 1996; Stamps, Mangel, & Phillips, 1998). Yet in some perennial ectotherms, geographic patterns or sexual differences in body size at maturity can be strongly modified at later stages via postmaturity growth and survival (Howard, 1981; Rutherford, 2004; Tracy, 1999). In colder and more seasonal climates, perennial ectotherms tend to grow more slowly but live longer than in milder climates. The form of the adult size*—*seasonality relationship would apparently be determined by the relative strength of these two opposite trends. Obviously, a pseudo‐Bergmann cline more likely arises when the increase in adult survival is “disproportional”, i.e. when the life‐time activity actually increases in seasonal climates instead of being just distributed over more but shorter seasons. Such an increase may occur if the survival increment caused by reduced lizards' potential annual activity (Adolph & Porter, 1993) is added with an extra increment caused by reduced predation and parasite pressure. A corresponding increase in SSD with increasing longevity is expectable if the post‐maturity growth is less decelerated in the larger sex, especially when the larger sex also exhibits a higher adult survival (Watkins, 1996). Both patterns were reported for *Zootoca vivipara* (growth: Epova et al., 2016; survival: Strijbosch & Creemers, 1988; Uller, Massot, Richard, Lecomte, & Clobert, 2004; Le Galliard et al., 2010). The presented mechanism may strengthen the overall pseudo‐Bergmann trend predicted by the Adolph & Porter (1996) model and explain the increase in female size and SSD with increasing seasonality revealed within the eastern viviparous clade and across the studied clades of *Z. vivipara* (Figure 2a,d). However, this mechanism cannot explain the opposing cline in the western viviparous clade.

The intriguingly disparate body size—climate relationships in the western and the eastern viviparous clades are difficult to explain outside the Adolph & Porter (1996) model. One alternative scenario would be that the western and eastern viviparous clades differ in their inherited thermal reaction norms (Angilletta, 2009) or potential for response to selection, thus being genetically predisposed to develop opposing body size clines along similar climatic gradients. Currently, such a scenario, implying a substantive role of intrinsic factors, seems unlikely considering that the two clades are closely related (Surget‐Groba et al., 2006) and show no appreciable differences in life‐history (Roitberg et al., 2013) and external morphology (Guillaume et al., 2006).
